# *NCK-associated protein 1 like (nckap1l*) minor splice variant regulates intrahepatic biliary network morphogenesis

**DOI:** 10.1371/journal.pgen.1009402

**Published:** 2021-03-19

**Authors:** Kimia Ghaffari, Lain X. Pierce, Maria Roufaeil, Isabel Gibson, Kevin Tae, Saswat Sahoo, James R. Cantrell, Olov Andersson, Jasmine Lau, Takuya F. Sakaguchi

**Affiliations:** 1 Department of Inflammation and Immunity, Lerner Research Institute, Cleveland Clinic, Cleveland, Ohio, United States of America; 2 Cleveland Clinic Lerner College of Medicine of Case Western Reserve University, Cleveland, Ohio, United States of America; 3 Department of Cell and Molecular Biology, Karolinska Institutet, Stockholm, Sweden; 4 Department of Molecular Medicine, Cleveland Clinic Lerner College of Medicine of Case Western Reserve University, Cleveland, Ohio, United States of America; University of Pennsylvania School of Medicine, UNITED STATES

## Abstract

Impaired formation of the intrahepatic biliary network leads to cholestatic liver diseases, which are frequently associated with autoimmune disorders. Using a chemical mutagenesis strategy in zebrafish combined with computational network analysis, we screened for novel genes involved in intrahepatic biliary network formation. We positionally cloned a mutation in the *nckap1l* gene, which encodes a cytoplasmic adaptor protein for the WAVE regulatory complex. The mutation is located in the last exon after the stop codon of the primary splice isoform, only disrupting a previously unannotated minor splice isoform, which indicates that the minor splice isoform is responsible for the intrahepatic biliary network phenotype. CRISPR/Cas9-mediated *nckap1l* deletion, which disrupts both the primary and minor isoforms, showed the same defects. In the liver of *nckap1l* mutant larvae, WAVE regulatory complex component proteins are degraded specifically in biliary epithelial cells, which line the intrahepatic biliary network, thus disrupting the actin organization of these cells. We further show that *nckap1l* genetically interacts with the Cdk5 pathway in biliary epithelial cells. These data together indicate that although *nckap1l* was previously considered to be a hematopoietic cell lineage-specific protein, its minor splice isoform acts in biliary epithelial cells to regulate intrahepatic biliary network formation.

## Author summary

NCK Associated Protein 1 Like (Nckap1l), a cytoplasmic adapter protein, was previously considered a hematopoietic cell linage-specific member of the WAVE regulatory complex. In this study, through a forward genetic screen in zebrafish (*Danio rerio*), we identified a mutation in *nckap1l* that leads to specific branching morphogenesis defects in the intrahepatic biliary network. The mutation specifically affects a novel short splice isoform of *nckap1l*, indicating that this short isoform is important for the branching morphogenesis phenotype. We found that the Nckap1l protein is expressed in biliary epithelial cells. We also show that the *nckap1l* mutant genetically interacts with the Cdk5 pathway to regulate actin dynamics in biliary epithelial cells. This study identified a previously unappreciated correlation between hematopoietic and biliary cell lineages: Nckap1 is known to regulate hematopoietic cell lineage differentiation but this study finds that its short splice isoform plays additional roles in biliary system formation.

## Introduction

The biliary system is responsible for transporting bile from the liver to the digestive tract. The biliary system within the liver, the intrahepatic biliary network, is a highly branched three-dimensional network that is found throughout the liver. Biliary epithelial cells, also called cholangiocytes, are the innermost epithelial cells forming the conduit of this network. Three-dimensional branching anomalies in the intrahepatic biliary network are implicated in many liver diseases, including biliary atresia [1,2]. Biliary atresia is the most common neonatal cholestatic disorder, occurring in approximately 1 of 18,000 live births [3], but the molecular etiology and pathogenesis of this disease are poorly understood. Biliary atresia is not generally thought of as an inherited disease; however, there are some lines of evidence to support the hypothesis that genetic factors contribute to susceptibility to this disease [4–6]. However, the genes and signaling pathways underlying genetic susceptibility to biliary atresia affecting intrahepatic biliary network branching morphogenesis have not been fully investigated. Dysregulation of the innate immune system has been proposed to be involved in the pathogenesis of biliary atresia [7]. However, the question of whether genes important for the innate immune system also affect biliary system formation has just begun to be investigated.

We have previously used a pharmacogenetics approach to show that the Cdk5-Pak1-Limk1-Cofillin kinase cascade regulates branching morphogenesis of the intrahepatic biliary network and actin dynamics in biliary epithelial cells [8]. However, the extent to which this Cdk5-mediated signaling pathway is linear or branched to regulate actin dynamics is not known.

Actin dynamics is accompanied by rapid site-directed nucleation and polymerization of actin into filaments [9], which is regulated by multiple regulatory factors, including the WASP-family verprolin-homologous protein (WAVE) regulatory complex [10,11]. The WAVE regulatory complex exists as a pentameric heterocomplex that consists of WAVE (WAVE1, WAVE2 or WAVE3), Abi (Abi1 or Abi2), Nap (Nap1 or NCK-associated protein 1-like (Nckap1l)), Sra (Sra1 or Sra2) and HSPC300 proteins [10]. Several upstream factors including the Rac1 GTPase [12–14] and Cyclin-dependent kinase 5 (Cdk5) [15] are known to regulate the activity of the WAVE regulatory complex to control actin dynamics. Nckap1l, which is also known as Hematopoietic protein-1 (Hem-1), is considered to be a hematopoietic cell lineage-specific member of the WAVE regulatory complex [16–18], as Nckap1l knockout mice show specific phenotypes in hematopoietic cell deployment and function [16]. However, Nckap1l functions outside of hematopoietic cells are not known.

Alternative splicing is a general regulatory mechanism that produces more than one mRNA isoform from a single gene, allowing the generation of different protein isoforms with diverse functions or localizations from a single gene [19]. Approximately 90–95% of human genes are known to undergo alternative splicing [20]; however, further work is required to uncover the physiological functions of each of those splice isoforms. Some cardiac and neuronal developmental human diseases are associated with errors in alternative splicing mechanisms [19]. However, it is not known whether any alternative splicing is involved in biliary system formation.

In this study, through N-ethylnitrosourea (ENU)-mediated forward genetic screening in zebrafish combined with computational intrahepatic biliary network structural analysis, we have identified a new mutation that genetically interacts with the Cdk5-mediated pathway during branching morphogenesis. We identified that the mutation disrupts a previously unannotated minor splice isoform of the *nckap1l* gene, and propose that this minor isoform of Nckap1l functions downstream of Cdk5 and Rac1 to regulate branching morphogenesis of the intrahepatic biliary network.

## Results

### A forward genetic screen identified the *lri35* allele showing a specific phenotype in the intrahepatic biliary network

**Fig 1:**
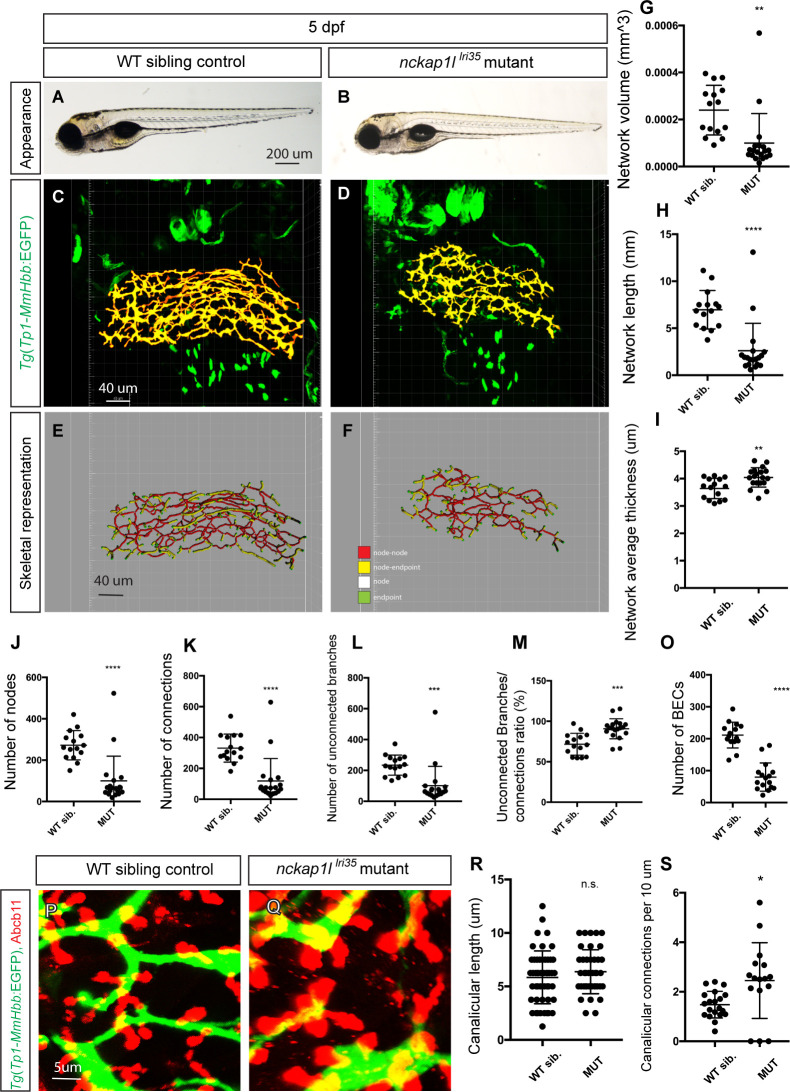
*nckap1l*^*lri35*^ mutant larvae show specific defects in the intrahepatic biliary network. (A and B) Representative physical appearance of wild-type (WT) sibling (A) and *nckap1l*^*lri35*^ mutant (B) larvae at 5 days post-fertilization (dpf). Lateral views. At 5 dpf, there is no significant difference in physical appearance in *nckap1l*^*lri35*^ mutant larvae, although their swim bladder inflation is occasionally delayed. (C and D) Projected confocal images of *Tg(Tp1-MmHbb*:EGFP*)*^*um14*^ expression in WT (C) and *nckap1l*^*lri35*^ mutant (D) larvae at 5 dpf. GFP expression in the intrahepatic biliary network is shown in pseudocolored yellow. Ventral views, anterior to the top. (E and F) Skeletal representation of the intrahepatic biliary network in WT (E) and *nckap1l*^*lri35*^ mutant (F) larvae computed based on *Tg(Tp1-MmHbb*:EGFP*)*^*um14*^ expression at 5 dpf. The complex three-dimensional network is represented by a combination of four segments: end points (colored green), nodes (colored white), node-node connections (colored red), and node-end point connections (colored yellow). (G-M) Computational skeletal analysis-based measurements of the intrahepatic biliary network structures of WT and *nckap1l*^*lri35*^ mutant larvae at 5 dpf. (G) The total network volume of the intrahepatic biliary network marked by *Tg(Tp1-MmHbb*:EGFP*)*^*um14*^ expression in the liver. (H) The total network length of the intrahepatic biliary network. (I) The average thickness of the intrahepatic biliary network. (J) The total number of nodes existing in the intrahepatic biliary network. (K) The total number of node-to-node connections. (L) The total number of unconnected branches (node-to-end point connections). (M) The ratio of connected to unconnected branches shown as a percentage. Each dot represents the measurement data from one larva. n = 13 for WT siblings and n = 17 for mutant larvae. Error bars are standard deviation. *P<0.05, **P<0.01. n.s., not significant. (N and O) Projected images of confocal z-stacks of the liver in WT (N) or *nckap1l*^*lri35*^ mutant (O) larvae visualized for expression of the bile canaliculi marker Abcb11 (Red) and the intrahepatic biliary network marker *Tg(Tp1-MmHbb*:EGFP*)*^*um14*^ (Green) at 5 dpf. (P) Average length of canaliculus measured based on Abcb11 expression. A total of 90 canaliculi were analyzed. (Q) The number of canaliculi connected per 10 μm of the intrahepatic biliary network (n = 50 for WT and n = 40 for *nckap1l*^*lri35*^ mutant). Error bars are standard deviation. *P<0.05. n.s., not significant.

In the course of ENU-based mutagenesis utilizing *Tg(Tp1-MmHbb*:EGFP*)*^*um14*^ expression [21,22] in the intrahepatic biliary network and its computational network structure analysis [8], we identified a recessive mutant, *lri35*, which shows a phenotype similar to that of Cdk5-suppressed larvae [8] (Materials and Methods). The *lri35* mutation is viable, and the physical appearance of the majority of *lri35* mutant larvae is almost indistinguishable from that of wild-type siblings (Fig 1A and 1B) at 5 days post-fertilization (dpf), while approximately 23% of *lri35* mutant larvae (11/47 mutant larvae from three independent crosses) show a delay in swim bladder inflation at this stage. At this stage, the body length of *lri35* mutant larvae (average 3.87 mm, s.d. = 0.20, n = 11) is not significantly different from that in wild-type siblings (average 3.96 mm, s.d. = 0.12, n = 14, p = 0.17) at 5 dpf [23], and the size of the liver (Materials and Methods) remains unchanged. However, at this stage, the intrahepatic biliary network in *lri35* mutant larvae appears to show fewer branches (Fig 1C and 1D). To quantify the difference in the branching pattern between wild-type and *lri35* mutant larvae, we utilized the computational network structure analysis and measured the structural properties of the intrahepatic biliary network (Fig 1E and 1F). We found that network volume (Fig 1G) and length (Fig 1H) in *lri35* mutant larvae are reduced while network average thickness is increased (Fig 1I), indicating that the intrahepatic biliary network becomes thicker and shorter in *lri35* mutant larvae. In *lri35* mutant larvae, the number of nodes (Fig 1J), node-node connections (Fig 1K), and unconnected branches (node-endpoint connections) (Fig 1L) are all reduced while the ratio of connected and unconnected branches is increased (Fig 1M), suggesting that new branches fail to form connections properly in *lri35* mutant larvae. The number of *Tg(Tp1-MmHbb*:EGFP*)*^*um14*^ expressing cells in the liver is reduced in *lri35* mutant larvae at 5 dpf (Fig 1O), suggesting that the number of BECs is reduced. Bile canaliculi are the apical membranes of hepatocytes that connect to the intrahepatic biliary network. In *lri35* mutant larvae, bile canaliculi, as marked by Abcb11 expression [8], remain relatively unaffected (Fig 1P and 1Q), and the average canalicular length is not changed (Fig 1R). The density of canalicular connection to the intrahepatic biliary network is increased in *lri35* mutant larvae (Fig 1S), possibly due to the reduction of the total network length of the intrahepatic biliary network. Overall, these data together indicate that the *lri35* mutation impacts branching morphogenesis of the intrahepatic biliary network in a relatively specific manner.

### The *lri35* mutation disrupts the minor splice isoform of *nckap1l*

**Fig 2:**
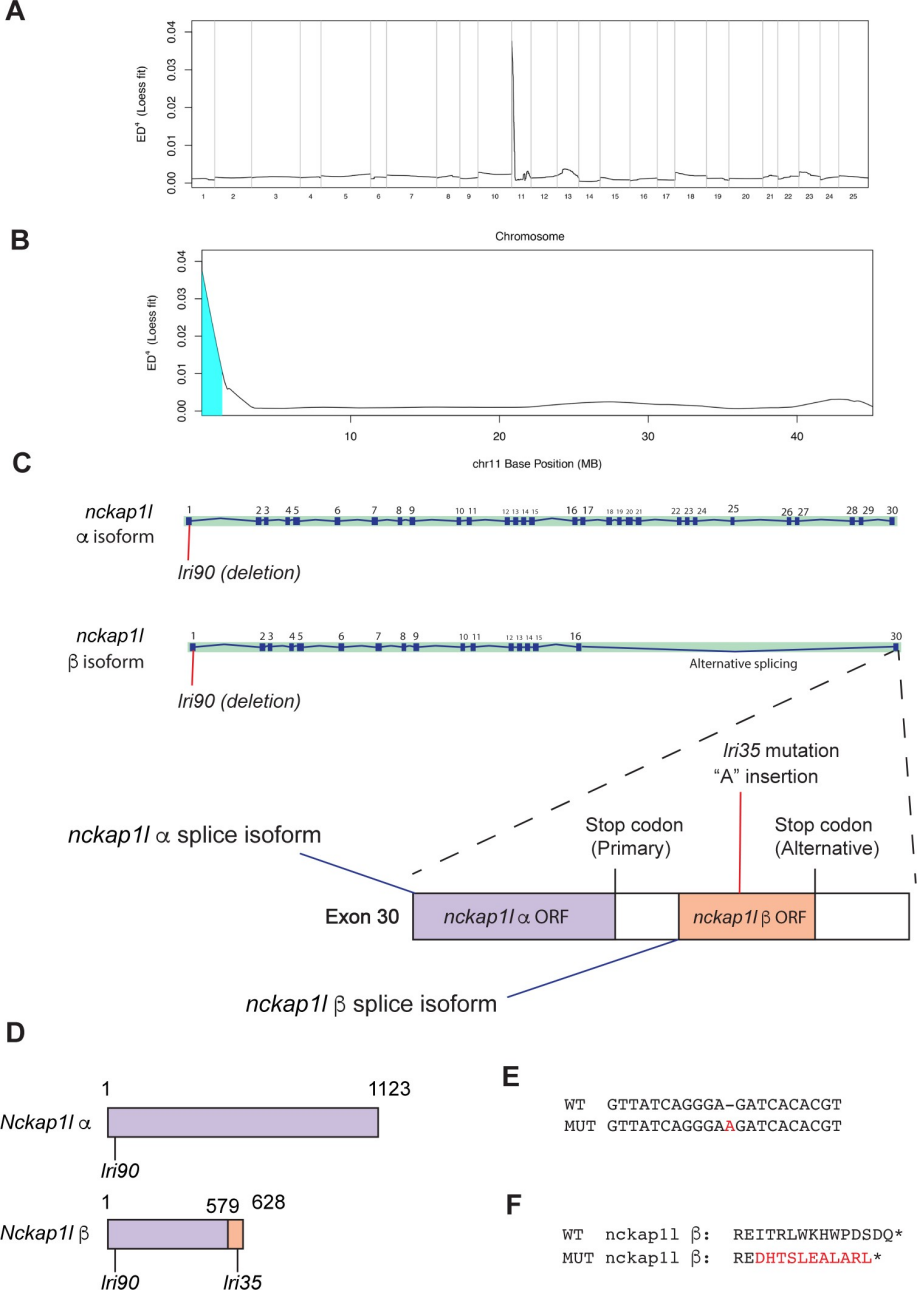
The *lri35* mutation disrupts the minor isoform of the *nckap1l* gene. (A) Mapping of the *lri35* mutation. Whole genome-wide Loess fit curve for SNP allele frequency Euclidean distance computed by the MMAPPR algorithm. Chromosome 11 showed the highest score. (B) Loess fit curve for SNP allele frequency Euclidean distance within chromosome 11. The critical region was mapped to the distal tip of chromosome 11. (C) The *nckap1l* gene has two splice isoforms: the previously annotated major splice isoform (α isoform) and an unknown minor isoform (ß isoform). The *lri35* mutation, which is a one-nucleotide insertion, is located in the last exon and induces a frameshift only in the ß isoform of the *nckap1l* gene. These data indicate that the minor isoform of the *nckap1l* gene is responsible for the phenotypes in *lri35* mutant larvae. (D) Schematic of Nckap1l α and ß proteins. The entire Nckap1l α protein is recognized as the Nckap1l domain (purple box). Due to the alternative splicing of the *nckap1l* gene, Nckap1l ß shifted to the ß specific sequence (orange box) after the position 579. The *lri90* mutation affects both Nckap1l α and ß, whereas the lri35 mutation affects Nckap1l ß only. (E) The *lri35* mutation is an insertion mutation that inserts an additional adenine nucleotide into the *nckap1l* gene. (F) The *lri35* mutation induces a frameshift and changes the last 13 amino acids of the ß isoform of the Nckap1l protein.

Until recently, mapping of a causative mutation of ENU mutagenesis-derived mutants was an extremely labor-intensive process. However, the development of next-generation sequencing-based approaches [24–26] has made this process significantly easier. We applied the MMAPPR algorithm [24] to RNA-seq data obtained from *lri35* mutant larvae at 5 dpf (Materials and Methods) to map the location of the causative mutation. The *lri35* mutation was mapped to chromosome 11 at a region closer to the distal tip (Fig 2A and 2B). Based on the RNA-seq data, we first examined whether there were any SNPs inducing a premature stop within the critical region, but we did not detect any of these mutations. We next screened for differential gene expression levels and differential exon usage of genes within the critical region by using the Cufflinks algorithm [27]. We found that the most down-regulated gene within the critical region (yet bigger than a half-fold change) in *lri35* mutant larvae was *nckap1l*. Based on these results, we hypothesized that the *lri35* mutation might disrupt the *nckap1l* gene. To test this hypothesis, we first injected antisense morpholino (MO) against *nckap1l* into wild-type *Tg(Tp1-MmHbb*:EGFP*)*^*um14*^ eggs (Materials and Methods). We found that at 5 dpf, larvae injected with *nckap1l* MO did not show any changes in physical appearance, but did show a specific phenotype in the intrahepatic biliary network (S1 Fig) consistent with the hypothesis that *nckap1l* is mutated in *lri35* mutant larvae. We next induced an indel mutation to the *nckap1l* gene in wild-type using CRISPR/Cas9-based genome editing technology [28,29]. We injected the assembled CRISPR/Cas9 complex targeting *nckap1* into fertilized zebrafish eggs (Materials and Methods) and subsequently established a deletion allele, *nckap1l*^*lri90*^, that deletes 7 bp from the first exon and induces a premature stop at the position of ^3^tyrosine (S2A–S2C Fig). In homozygous *nckap1l*^*lri90*^ mutant larvae at 5 dpf, physical appearance is not affected (S2D and S2E Fig), but the intrahepatic biliary network shows lower three-dimensional-branching complexity (S2F–S2I Fig). Computed network structural sub-parameters of *nckap1l*^*lri90*^ mutant larvae are significantly different from those of WT control larvae and are similar to those of *lri35* mutant larvae (S2J–S2R Fig). *nckap1l*^*lri90*^ mutant larvae show a similar phenotype (S2 Fig) to that of *lri35* mutant larvae, suggesting that these two mutations disrupt the same gene. At 5 dpf, *nckap1l*^*lri90*^ mutant larvae also show functional defects in their biliary system, as indicated by reduced PED6 trafficking to the gallbladder [8,30] (S2S Fig). The *nckap1l*^*lri90*^ mutation is viable, and homozygous *nckap1l*^*lri90*^ adult fish show no overt difference in physical appearance. However, the *Tg(Tp1-MmHbb*:EGFP*)*^*um14*^ positive network of the homozygous *nckap1l*^*lri90*^ adult liver appears to be thinner and less dense (S2U Fig) than that of wild-type fish, suggesting that the mutation continues to affect the intrahepatic biliary network in the adult stage. To test the hypothesis that the *lri35* mutation disrupts the *nckap1l* gene, we attempted a genetic complementation approach by crossing heterozygous *lri35* and homozygous *nckap1l*^*lri90*^ mutant fish. We found that approximately 50% (20/43) of the larvae from this cross show a phenotype indistinguishable from either that of *lri35* or *nckap1l*^*lri90*^ mutant larvae (S3 Fig), indicating that the *lri35* allele failed to complement the *nckap1l*^*lri90*^ allele and that these two mutations disrupt the same gene. However, to our surprise, we did not find any mutation when we sequenced the *nckap1l* cDNA isolated from *lri35* mutant larvae at 5 dpf (Fig 2C). We also sequenced most of the annotated exon/intron boundaries of the *nckap1l* gene in *lri35* mutant larvae but did not find any mutations. By chance during these intensive sequence attempts, we found a previously unannotated splice isoform of *nckap1l*, which we refer to as *nckap1l ß*. This alternative splice variant skips all exons after exon 16 and uses an alternative stop codon in the last exon (Fig 2C). Thus, the encoded Nckap1l ß protein is smaller than Nckap1l (Fig 2D). We isolated the *nckap1l ß* cDNA from *lri35* mutant larvae and found that one adenine nucleotide is inserted into the ß-specific coding region (Figs 2C, 2E and S4). This insertion changes the last 13 amino acids of the Nckap1l ß protein (Fig 2F). We measured relative *nckap1l α* and *ß* gene expression in *lri35* mutant larvae at 5 dpf by quantitative RT-PCR and found that *nckap1l ß* expression is slightly reduced in *lri35* mutant larvae whereas *nckap1l α* expression remains unchanged (S5A and S5B Fig). Since the entire *nckap1l ß* gene sequence is part of the ORF and UTR of the *nckap1l* α gene, we were not able to design the *nckap1l ß*-specific RNA probe for *in situ* hybridization. Instead, we compared *nckap1l* α-specific probe to *nckap1l* α and *ß* shared probe, and found that the *nckap1l* α and *ß* shared probe stained the liver more strongly than the *nckap1l* α-specific probe, suggesting that *nckap1l ß* might be expressed more than *nckap1l* α in the liver at 5 dpf (S5C and S5D Fig). Based on these data, we propose that the *lri35* mutation is an adenine nucleotide insertion that specifically disrupts the ß isoform of the *nckap1l* gene, indicating that this minor splice isoform of *nckap1l* is important for intrahepatic biliary network branching morphogenesis.

### Nckap1l is expressed in hepatic biliary epithelial cells

**Fig 3:**
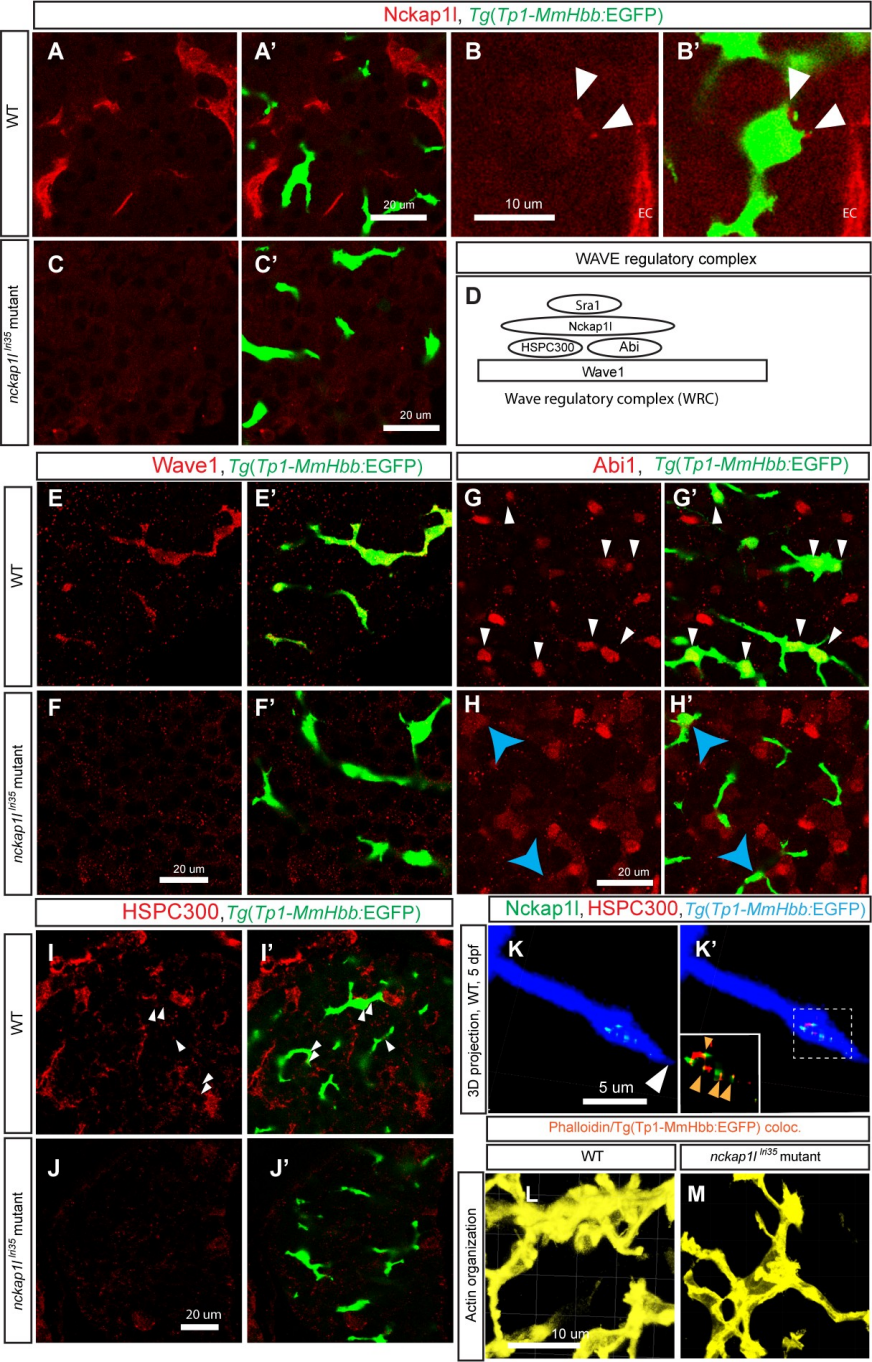
Expression of Nckap1l and other WRC proteins in the liver of wild-type and *nckap1l*^*lri35*^ mutant larvae. (A-C) Z-plane confocal images of the liver visualized for Nckap1l (Red) expression in wild-type (WT) (A and B) and *nckap1l*^*lri35*^ mutant (C) larvae at 5 dpf. Overlay images with biliary epithelial cell marker *Tg(Tp1-MmHbb*:EGFP*)*^*um14*^ (Green) shown separately in (A’-C’). Nckap1l is predominantly expressed in endothelial cells in the liver (A), but higher magnification z-plane image (B) shows that Nckap1l is also localized in biliary epithelial cells (white arrowheads). Nckap1l expression is missing in the liver of *nckap1l*^*lri35*^ mutant larvae (C). Ventral views, anterior to the top. EC, endothelial cells. (D-H) Components of the WAVE regulatory complex are degraded in biliary epithelial cells of *nckap1l*^*lri35*^ mutant larvae. (D) Schematic drawing of the WAVE regulatory complex (WRC). This complex acts downstream of Cdk5 and activated Rac1 to stimulate actin remodeling through the actin regulatory complex. The WAVE regulatory complex is a pentameric heterocomplex that consists of WAVE (1, 2 or 3), Abi (1 or 2), Sra1, Nckap1l (or Nap1), and HSPC300. (E and F) Z-plane confocal images of the liver visualized for WAVE1 expression (Red) in WT (E) and *nckap1l*^*lri35*^ mutant (F) larvae at 5 dpf. Overlay images with biliary epithelial cell marker *Tg(Tp1-MmHbb*:EGFP*)*^*um14*^ are shown separately in (E’ and F’). WAVE1 is expressed predominantly in biliary epithelial cells in WT larvae; however, in *nckap1l*^*lri35*^ mutant larvae, WAVE1 expression disappears from biliary epithelial cells, suggesting that WAVE1 undergoes degradation in biliary epithelial cells. (G and H) Z-plane confocal images of the liver visualized for Abi1 expression in WT (G) and *nckap1l*^*lri35*^ mutant (H) larvae at 5 dpf. Overlay images with biliary epithelial cell marker *Tg(Tp1-MmHbb*:EGFP*)*^*um14*^ are shown separately in (G’ and H’). Abi1 localizes to the nuclei of hepatocytes and biliary epithelial cells (white arrowheads) in the wild-type liver. However, in the liver of *nckap1l*^*lri35*^ mutant larvae, Abi1 staining in the nuclei of biliary epithelial cells is lost (yellow arrows), while Abi1 expression remains in hepatocytes, suggesting that Abi1 undergoes degradation specifically in biliary epithelial cells. (I and J) Z-plane confocal images of the liver visualized for HSPC300 expression in WT (I) and *nckap1l*^*lri35*^ mutant (J) larvae at 5 dpf. Overlay images with biliary epithelial cell marker *Tg(Tp1-MmHbb*:EGFP*)*^*um14*^ are shown separately in (I’ and J’). Similar to Nckap1l, HSPC300 is predominantly expressed in endothelial cells in the liver, but HSPC300 is also expressed in biliary epithelial cells (White arrowheads in I). HSPC300 expression is missing in the liver of *nckap1l*^*lri35*^ mutant larvae (J). (K) Projected confocal image of co-localizing signal between Nckap1l and *Tg(Tp1-MmHbb*:EGFP*)*^*um14*^ showing Nckap1l expression (pseudocolored green) in biliary epithelial cells at 5 dpf. *Tg(Tp1-MmHbb*:EGFP*)*^*um14*^ expression is shown in pseudocolored blue. Nckap1l is localized near the projection tip (white arrowhead), but not exactly at the tip. Colocalizing signal between HSPC300 and *Tg(Tp1-MmHbb*:EGFP*)*^*um14*^ showing HSPC300 expression (red) in biliary epithelial cells is overlaid in (K’). This indicates that HSPC300 is expressed in biliary epithelial cells. Nckap1l and HSPC300 colocalize in biliary epithelial cells (orange arrowheads) near the projection tips of biliary epithelial cells, suggesting that the WRC forms at this site. Boxed area is magnified and shown separately in the left bottom corner. A representing image of total 21 projection tips from 5 different wild-type larvae examined is shown. Ventral views, anterior to the top. (L and M) Projected co-localizing signal images computed based on *Tg(Tp1-MmHbb*:EGFP*)*^*um14*^ and phalloidin expressions visualizing the actin network in biliary epithelial cells in wild-type (I) and *nckap1l*^*lri35*^ mutant (J) larvae at 5 dpf. Confocal images are representative of at least three independent experiments.

In mice, Nckap1l is predominantly expressed in immune cells [16]; however, its expression within biliary epithelial cells has not been studied. We generated an antibody against zebrafish Nckap1l (Materials and Methods) and examined Nckap1l expression in the zebrafish larval liver at 5 dpf. We found that Nckap1l is predominantly expressed in vascular endothelial cells (Figs 3A and S6). We also observed small puncta of Nckap1l expression in *Tg(Tp1-MmHbb*:EGFP*)*^*um14*^-positive biliary epithelial cells (Fig 3B). Expression in both endothelial and biliary epithelial cells is missing in *nckap1l*^*lri35*^ mutant larvae at 5 dpf (Figs 3C and S7), suggesting that the Nckap1l protein is degraded in the mutant larvae.

### WAVE regulatory complex component proteins are degraded in biliary epithelial cells in the liver of *nckap1l*^*lri35*^ mutant larvae

In other systems, depletion of Nckap1l leads to the destruction of other WAVE regulatory complex (WRC) component proteins (Fig 3D), including WAVE and Abi1, without influencing their level of gene expression [16,18,31–33]. We found that WAVE1 is expressed predominantly in biliary epithelial cells in the liver of wild-type larvae at 5 dpf (Fig 3E), and this WAVE1 expression is decreased in *nckap1l*^*lri35*^ mutant larvae (Fig 3F). Moreover, in the wild-type liver at 5 dpf, consistent with a previous report that Abi1 undergoes nucleocytoplasmic shuttling [34], Abi1 is localized to the nucleus of some hepatocytes and biliary epithelial cells (Fig 3G). In *nckap1l*^*lri35*^ mutant larvae, Abi1 expression in biliary epithelial cells is specifically missing, while its expression in hepatocytes remains unchanged (Fig 3H). Similarly, we found that HSPC300 is expressed in the liver, including in biliary epithelial cells, of wild-type larvae at 5 dpf (Fig 3I), and this HSPC300 expression is decreased in *nckap1l*^*lri35*^ mutant larvae (Fig 3J). Three-dimensional projected confocal images of the liver in wild-type larvae indicated that Nckap1l and HSPC300 colocalize in biliary epithelial cells near the projection tips of biliary epithelial cells (Fig 3K) at 5 dpf, suggesting that the WRC forms at this site. These data together indicate that WRC forms in biliary epithelia cells in wild-type larvae, and WRC component proteins are degraded in the liver of *nckap1l*^*lri35*^ mutant larvae.

Consistent with the observation that WAVE regulatory complex proteins are degraded in biliary epithelial cells, the actin organization of *nckap1l*^*lri35*^ mutant larvae changes and becomes concentrated around the lumen of biliary epithelial cells (Fig 3L and 3M).

### Overexpression of *nckap1l ß* specifically impacts the intrahepatic biliary network

**Fig 4:**
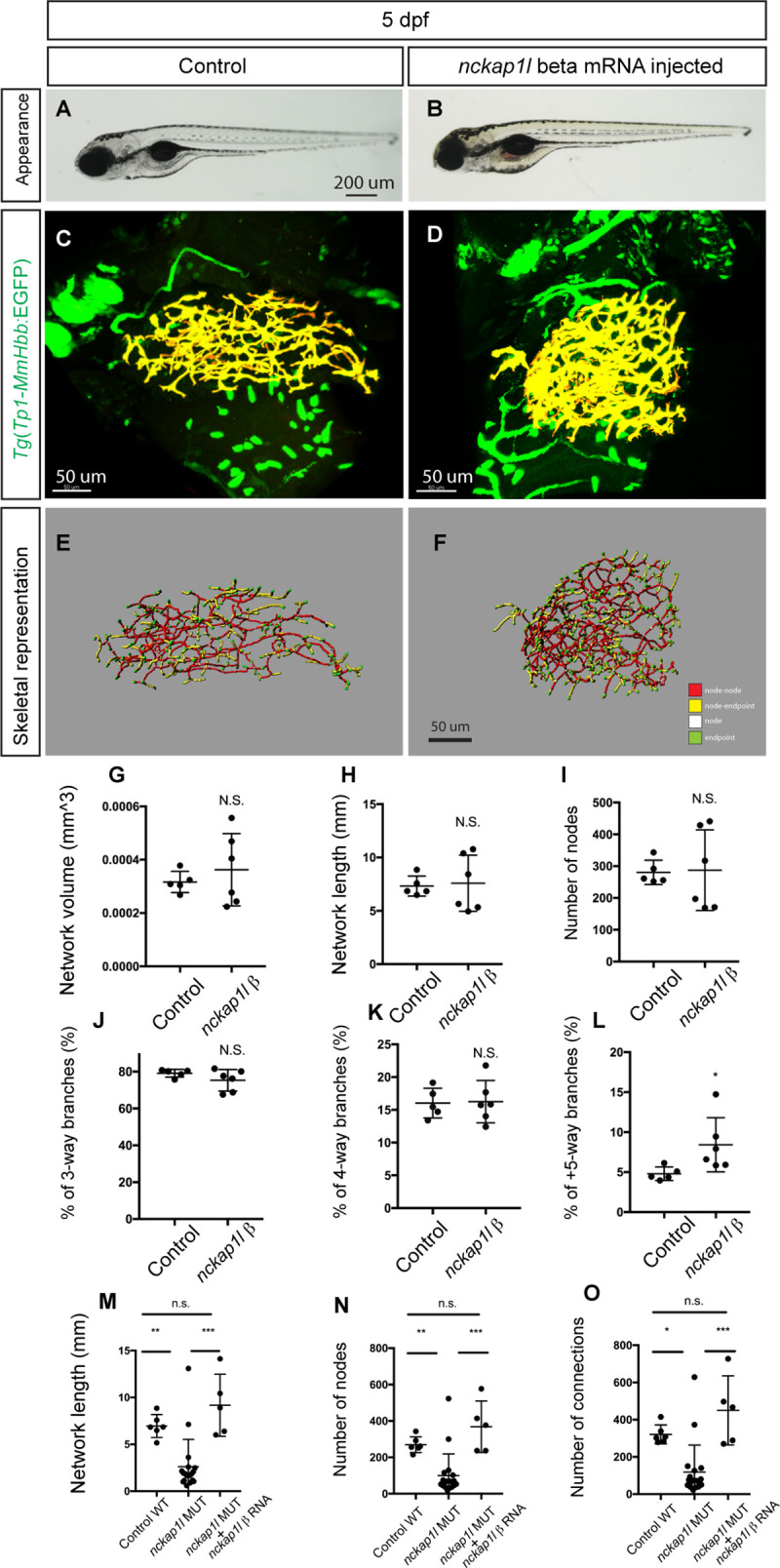
*nckap1l* ß overexpression induced biliary system-specific phenotypes. (A and B) Representative physical appearance of wild-type (WT) control (A) and *nckap1l* ß mRNA-injected (B) larvae at 5 dpf. Lateral views. At 5 dpf, there is no significant difference in physical appearance in larvae overexpressing *nckap1l* ß. (C and D) Projected confocal images of *Tg(Tp1-MmHbb*:EGFP*)*^*um14*^ expression in WT (C) and *nckap1l* ß RNA-injected (D) larvae at 5 dpf. GFP expression in the intrahepatic biliary network is shown in pseudocolored yellow. (E and F) Skeletal representation of the intrahepatic biliary network in WT (E) and *nckap1l* ß RNA-injected (F) larvae computed based on *Tg(Tp1-MmHbb*:EGFP*)*^*um14*^ expression at 5 dpf. The end points (green), nodes (white), node-node connections (red), and node-end point connections (yellow) are colored separately. Ventral views, anterior to the top. (G-L) Computational skeletal analysis-based measurements of the intrahepatic biliary network structures of WT and *nckap1l* ß RNA-injected larvae at 5 dpf. (G) The total network volume of the intrahepatic biliary network marked by *Tg(Tp1-MmHbb*:EGFP*)*^*um14*^ expression in the liver. (H) The total network length of the intrahepatic biliary network. (I) The number of nodes in the intrahepatic biliary network. (J) The ratio of 3-way branching nodes per all nodes shown as a percentage. (K) The ratio of 4-way branching nodes per all nodes shown as a percentage. (L) The ratio of 5-or-more-way branching nodes per all nodes shown as a percentage. These data together indicate that *nckap1l* ß RNA injection induced a phenotype in the intrahepatic biliary network. n = 5 for WT, and n = 6 for *nckap1l* ß RNA-injected larvae. (M-O) *nckap1l* ß RNA injection rescued *nckap1l*^*lri35*^ mutant phenotypes. Network structural sub-parameters were calculated in uninjected *nckap1l*^*lri35*^ mutant, uninjected wild-type, and *nckap1l* ß RNA-injected *nckap1l*^*lri35*^ mutant larvae at 5 dpf. (M) The total network length. (N) The number of nodes. (O) The number of connections. *P<0.05, **P<0.01, and ***P<0.001. n.s., not significant.

As mutations in the *nckap1l ß* gene were found to impact intrahepatic biliary network formation, we next examined whether overexpression of *nckap1l ß* would also influence intrahepatic network formation. We synthesized the *nckap1l ß* mRNA *in vitro* (Materials and Methods) and injected it into *Tg(Tp1-MmHbb*:EGFP*)*^*um14*^ eggs. At 5 dpf, we did not observe any difference in physical appearance in *nckap1l* ß mRNA-injected larvae (Fig 4A and 4B). We found that all injected larvae appeared to show differences in intrahepatic biliary network branching pattern (Fig 4C–4F). However, likely due to the degradation of injected RNA by 5 dpf, there was high variability of the intrahepatic biliary network structural properties in larvae injected with *nckap1l* ß mRNA at 5 dpf (Fig 4G–4L), and only the percentage of 5-or-more-way branches among the entire network was increased significantly (Fig 4L), suggesting that the network in *nckap1l* ß mRNA-injected larvae contains nodes with more branches. These data indicate that overexpression of *nckap1l* ß can induce a relatively specific phenotype in the intrahepatic biliary network.

### Overexpression of *nckap1l* ß rescued *nckap1l*^*lri35*^ mutant phenotypes

We next injected *nckap1l* ß mRNA into eggs obtained from an *nckap1l*^*lri35*^ heterozygous fish intercross, and at 5 dpf, we genotyped homozygous *nckap1l*^*lri35*^ larvae injected with the *nckap1l* ß mRNA and examined intrahepatic biliary network formation. We found that network structural sub-parameters observed in *nckap1l*^*lri35*^ mutant larvae, including the total network length (Fig 4M), the numbers of nodes (Fig 4N) and connections (Fig 4O), are rescued by injecting *nckap1l* ß mRNA. These data indicate that supplying *nckap1l* ß mRNA can rescue, at least in part, intrahepatic biliary phenotypes in *nckap1l*^*lri35*^ mutant larvae, further confirming that the *nckap1l* ß gene is responsible for this mutation.

### *nckap1l* genetically interacts with Cdk5 and Rac1 during intrahepatic biliary network branching morphogenesis

**Fig 5:**
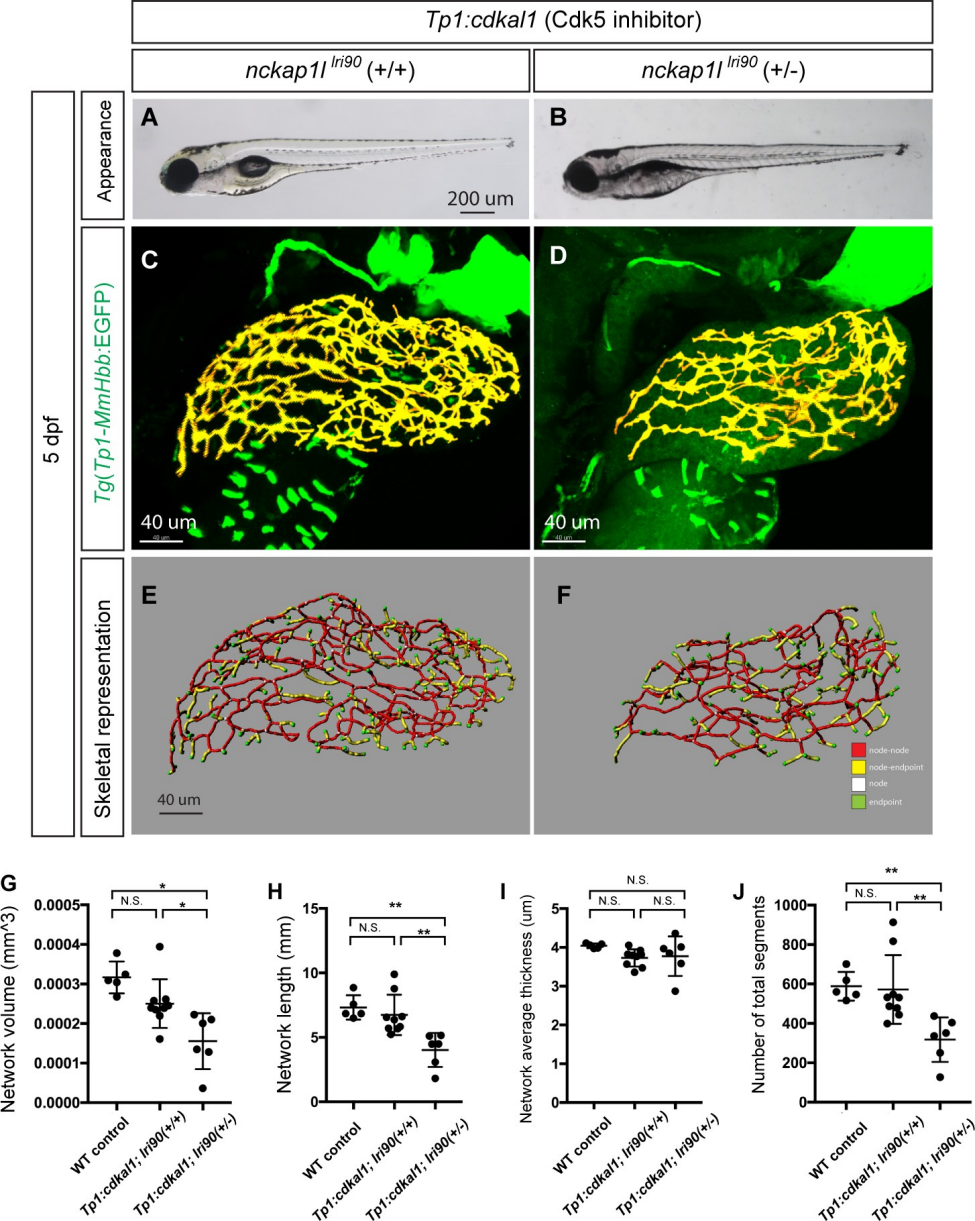
Dosage-sensitive genetic interaction between *nckap1l* and *cdk5* indicates these two genes function as part of the same pathway. (A and B) Representative physical appearance of *Tg(tp1*:*cdkal1)*^*kl109*^ larvae (*nckap1l*^*lri90(+/+)*^;*Tg(tp1*:*cdkal1)*^*kl109*^) (A), which expresses the Cdk5 inhibitor in biliary epithelial cells, and heterozygous *nckap1l*^*lri90*^ larvae expressing *Tg(tp1*:*cdkal1)*^*kl109*^ (*nckap1l*^*lri90(+/-)*^;*Tg(tp1*:*cdkal1)*^*kl109*^) (B) at 5 dpf. At 5 dpf, there is no significant difference in physical appearance in both genotypes. (C and D) Projected confocal images of *Tg(Tp1-MmHbb*:EGFP*)*^*um14*^ expression in *nckap1l*^*lri90(+/+)*^;*Tg(tp1*:*cdkal1)*^*kl109*^ (C) and *nckap1l*^*lri90(+/-)*^;*Tg(tp1*:*cdkal1)*^*kl109*^ (D) larvae at 5 dpf. GFP expression in the intrahepatic biliary network is shown in pseudocolored yellow. *nckap1l*^*lri90(+/-)*^;*Tg(tp1*:*cdkal1)*^*kl109*^ larvae show more severe intrahepatic biliary network phenotypes than *nckap1l*^*lri90(+/-)*^;*Tg(tp1*:*cdkal1)*^*kl109*^ larvae, although heterozygous *nckap1l*^*lri90*^ larvae show no observable phenotype without being crossed to *Tg(tp1*:*cdkal1)*^*kl109*^. (E and F) Skeletal representation of the intrahepatic biliary network in *nckap1l*^*lri90(+/+)*^;*Tg(tp1*:*cdkal1)*^*kl109*^ (E) and *nckap1l*^*lri90(+/-)*^;*Tg(tp1*:*cdkal1)*^*kl109*^ (F) larvae computed based on *Tg(Tp1-MmHbb*:EGFP*)*^*um14*^ expression at 5 dpf. The end points (green), nodes (white), node-node connections (red), and node-end point connections (yellow) are colored separately. Ventral views, anterior to the top. (G-J) Computational analysis-based measurements of the intrahepatic biliary network structures of wild-type (WT) control, *nckap1l*^*lri90(+/+)*^;*Tg(tp1*:*cdkal1)*^*kl109*^ and *nckap1l*^*lri90(+/-)*^;*Tg(tp1*:*cdkal1)*^*kl109*^ larvae at 5 dpf. n = 10 for *nckap1l*^*lri90(+/+)*^;*Tg(tp1*:*cdkal1)*^*kl109*^ and n = 7 for *nckap1l*^*lri90(+/-)*^;*Tg(tp1*:*cdkal1)*^*kl109*^. (G) The total network volume of the intrahepatic biliary network marked by *Tg(Tp1-MmHbb*:EGFP*)*^*um14*^ expression in the liver. (H) The total network length of the intrahepatic biliary network. (I) The average thickness of the intrahepatic biliary network. (J) Total segment number of the skeletonized network. These data together indicate that losing one copy of the *nckap1l* gene significantly enhanced the effect of Cdk5 suppression in biliary epithelial cells, suggesting that *nckap1l* and *cdk5* function in the same signaling pathway. *P<0.05, and **P<0.01. n.s., not significant.

**Fig 6:**
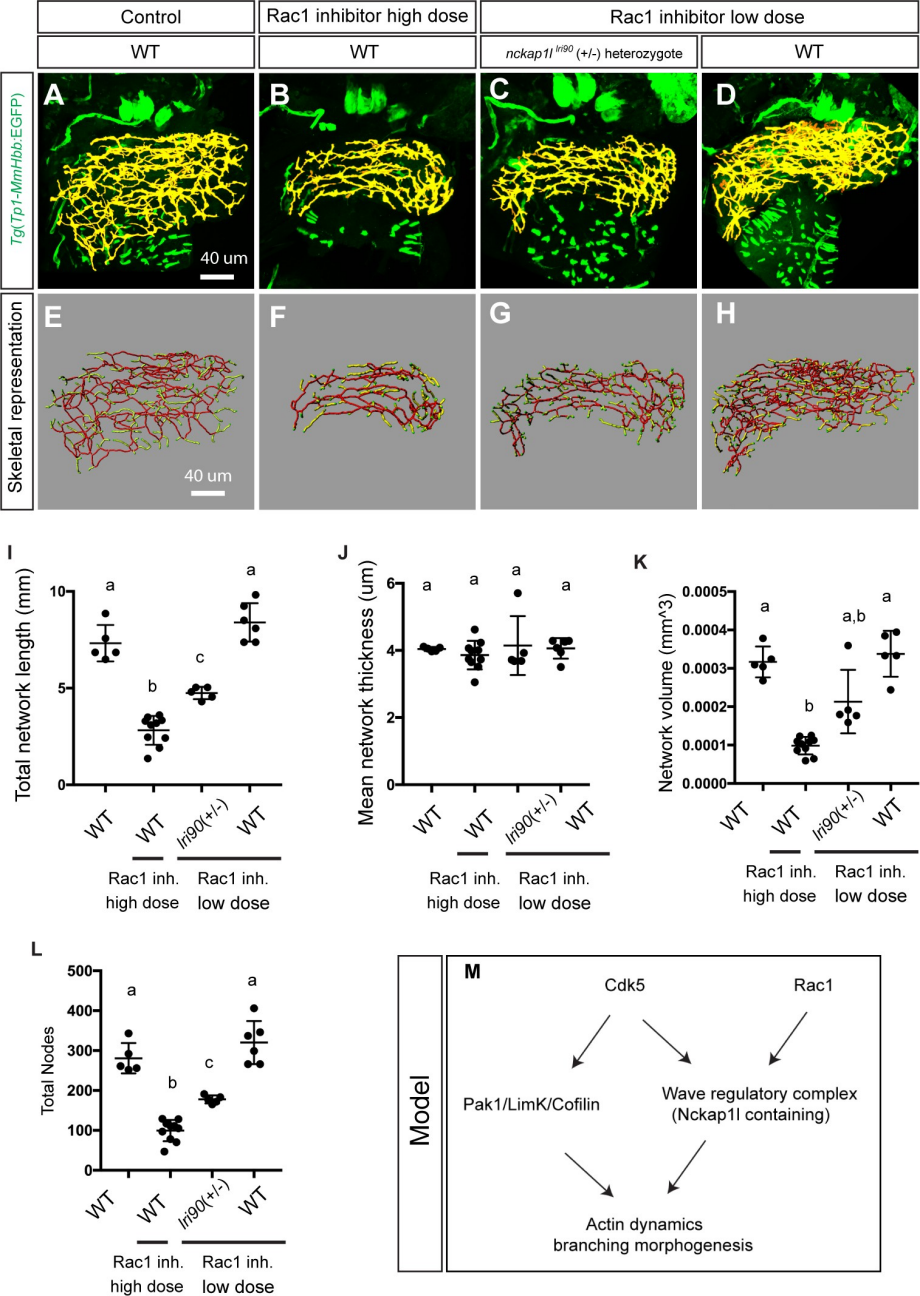
*nckap1l* dosage-dependent sensitization to Rac1 inhibitor treatment suggests that Nckap1l acts downstream of Rac1 to regulate intrahepatic biliary network branching morphogenesis. Wild-type and heterozygous *nckap1l*^*lri90*^ mutant larvae were treated with either high dose (50 ug/ml) or low dose (10 ug/ml) Rac1 inhibitor from 3 to 5 dpf, and then the treated larvae were analyzed at 5 dpf. (A-D) Projected confocal images of *Tg(Tp1-MmHbb*:EGFP*)*^*um14*^ expression in control wild-type (A), high dose (50 ug/ml) Rac1 inhibitor-treated wild-type (B), low dose (10 ug/ml) Rac1 inhibitor-treated heterozygous *nckap1l*^*lri90*^ mutant (C), and low dose (10 ug/ml) Rac1 inhibitor-treated wild-type (D) larvae at 5 dpf. GFP expression in the intrahepatic biliary network is shown in pseudocolored yellow. (E-H) Skeletal representation of the intrahepatic biliary network in wild-type (E), 50 ug/ml Rac1 inhibitor-treated wild-type (F), 10 ug/ml Rac1 inhibitor-treated heterozygous *nckap1l*^*lri90*^ mutant (G), and 10 ug/ml Rac1 inhibitor-treated wild-type (H) larvae computed based on *Tg(Tp1-MmHbb*:EGFP*)*^*um14*^ expression at 5 dpf. The end points (green), nodes (white), node-node connections (red), and node-end point connections (yellow) are colored separately. Ventral views, anterior to the top. (I-L) Computational analysis-based measurements of the intrahepatic biliary network structures of wild-type, high dose (50 ug/ml) Rac1 inhibitor-treated wild-type, low dose (10 ug/ml) Rac1 inhibitor-treated heterozygous *nckap1l*^*lri90*^ mutant, and low dose (10 ug/ml) Rac1 inhibitor-treated wild-type larvae at 5 dpf. (I) Total network length of the intrahepatic biliary network. (J) Mean network thickness of the intrahepatic network. (K) Network volume (mm^3^) of the intrahepatic biliary network. (L) Total number of nodes in the intrahepatic biliary network. These data together indicate that low dose (10 ug/ml) Rac1 inhibitor treatment did not cause any observable phenotype in the intrahepatic biliary network of wild-type larvae, but losing one copy of the *nckap1l* gene significantly enhanced the effect of low dose (10 ug/ml) Rac1 inhibitor treatment and induced a phenotype to closer to that of high dose (50 ug/ml) Rac1 inhibitor treated wild-type larvae. These data suggest that *nckap1l* acts downstream of Rac1 to regulate intrahepatic biliary network branching morphogenesis. Plots with a shared letter indicate that the difference is not statistically significant. (M) Model of the Cdk5-mediated kinase cascade that regulates branching morphogenesis of the intrahepatic biliary network. We have previously shown that Cdk5 regulates the Pak1/Limk1/Cofilin kinase cascade to regulate actin dynamics. The current study revealed that Cdk5 also regulates the WAVE regulatory complex to regulate branching morphogenesis of the intrahepatic biliary network. In this process, a previously unannotated minor splice isoform of Nckap1l appears to be important to form a functional WAVE regulatory complex in biliary epithelial cells.

We identified the *nckap1l*^*lri35*^ mutant as having a similar phenotype to that of Cdk5-suppressed larvae. In the mammalian nervous system, Cdk5 is known to directly phosphorylate WAVE1 to regulate actin cytoskeletal organization [15]. Thus, we hypothesized that *nckap1l* and *cdk5* might act in the same signaling pathway to regulate intrahepatic biliary network branching morphogenesis. To test this hypothesis, we examined the genetic interaction between *nckap1l* and *cdk5* in biliary epithelial cells. In the liver of *Tg(tp1*:*cdkal1)*^*kl109*^ larvae, the endogenous inhibitor of Cdk5 is expressed specifically in biliary epithelial cells [35]. Consistent with a previous report that the phenotype of *Tg(tp1*:*cdkal1)*^*kl109*^ larvae is very mild [35], we found that the branching pattern of the intrahepatic biliary network in *Tg(tp1*:*cdkal1)*^*kl109*^ larvae is not significantly changed from that of wild-type larvae at 5 dpf (Fig 5A, 5C and 5E). As the *nckap1l*^*lri90*^ mutation is recessive, we did not observe any phenotype in heterozygous *nckap1l*^*lri90*^ mutant animals. However interetingly, when we crossed heterozygous *nckap1l*^*lri90*^ mutant fish to homozygous *Tg(tp1*:*cdkal1)*^*kl109*^ fish, we found that approximately 50% of their offspring (n = 23/45 tested) showed a significantly more severe phenotype in the intrahepatic biliary network compared to that of *Tg(tp1*:*cdkal1)*^*kl109*^ larvae at 5 dpf (Fig 5B, 5D and 5F). This result strongly suggests that *nckap1l* and *cdk5* genetically interact in biliary epithelial cells. Subsequent genotyping confirmed that those 23 larvae showing an enhanced phenotype are all heterozygous *nckap1l*^*lri90*^ larvae expressing *Tg(tp1*:*cdkal1)*^*kl109*^, confirming that losing one copy of *nckap1l* in the *Tg(tp1*:*cdkal1)*^*kl109*^ background synergistically generates the phenotype via the dosage-sensitive genetic interaction. Computational network structure analysis confirmed that network volume, network length, and segment number are significantly reduced in heterozygous *nckap1l*^*lri90*^; *Tg(tp1*:*cdkal1)*^*kl109*^ larvae compared to those in *Tg(tp1*:*cdkal1)*^*kl109*^ larvae (Fig 5G–5J). These data together indicate that *nckap1l* genetically interacts with the *Cdk5* pathway in biliary epithelial cells to regulate intrahepatic biliary network remodeling. Finally, since the WRC is known to acts downstream of Rac1 [12–14,36], we tested whether the *nckap1l* mutation genetically interacts with the Rac1 pathway. Consistent with a previous report [37], 50 ug/ml Rac1 inhibitor treatment from 3 to 5 dpf did not induce any physical appearance change, but we found that the intrahepatic biliary network branching pattern was severely altered (Fig 6B and 6F). When we lowered the Rac1 inhibitor concentration to 10 ug/ml, it no longer affected intrahepatic biliary network branching patterns in wild-type larvae (Fig 6D and 6H). However, when we treated larvae obtained from *nckap1l*^*lri90*^ heterozygous outcross to wild-type fish from 3 to 5 dpf with the same low dose 10 ug/ml Rac1 inhibitor, approximately 50% (24/51) larvae showed a significantly more severe phenotype in the intrahepatic biliary network (Fig 6C, 6G, and 6I–6L), and we identified that these affected larvae are all heterozygous *nckap1l*^*lri90*^ larvae. These data together show that losing one copy of *nckap1l* sensitized the effects of the low dose Rac1 inhibitor, suggesting that *nckap1l* acts downstream of the Rac1 pathway.

In this study, through a forward genetic approach, we have identified Nckap1l as a novel factor regulating branching morphogenesis of the intrahepatic biliary network. Nckap1l is a component of the WAVE regulatory complex (WRC), which is known to act downstream of Cdk5 and Rac1 to regulate actin dynamics. Consistently, we show that the *nckap1l* mutation genetically interacts with the Cdk5 (Fig 5) and Rac1 (Fig 6) pathways in biliary epithelial cells, suggesting that we have identified a new signaling branch that acts downstream of Cdk5 and Rac1 to orchestrate branching morphogenesis of the intrahepatic biliary network (Fig 6M).

We initially focused on the *nckap1l*^*lri35*^ mutant phenotype even before identifying the responsible gene because the biliary phenotype calculated by the computational network structure analysis is similar to those of Cdk5-suppressed larvae [8]. It is intriguing that Nckap1l is a component of the WRC, which is directly phosphorylated by Cdk5 in other systems, and we have identified that the *nckap1l*^*lri35*^ mutation actually interacts genetically with the Cdk5 pathway (Fig 5). These data suggest that precise quantification of mutant phenotypes with our computational analysis could predict the signaling pathway in which the mutated gene lies.

In biliary epithelial cells, Nckap1l exhibits a punctate localization near the plasma membrane (Fig 3B). The projected confocal images indicated that Nckap1l colocalizes with HSPC300 near the tip of the projecting intrahepatic biliary network (Fig 3K), indicating that the WRC forms near the leading edge of protruding BECs to regulate branching morphogenesis, which is similar to the previous observation that the WRC is known to localize to the leading edges of lamellipodia in the migrating cell [18]. We previously showed the tight correlation between the actin organization in BECs and the intrahepatic biliary network branching pattern [8], in which the consolidated actin organization in BECs correlates with the paucity of intrahepatic biliary network branching. Consistent with this previous observation, we found that actin dynamics in BECs are consolidated in *nckap1l*^*lri35*^ mutant larvae (Fig 3L and 3M), suggesting that changes in BEC actin dynamics might influence the branching pattern of the intrahepatic biliary network.

In this study, we have identified a previously unannotated splice isoform of *nckap1l* in zebrafish, and the *nckap1l*^*lri35*^ mutation specifically affects this minor splice isoform (Fig 2); this study added a new gene to the list that previously shown to regulate intrahepatic biliary network formation in zebrafish [38–48]. As the *nckap1l*^*lri35*^ mutation does not affect the coding sequence of the major isoform (α) of *nckap1l*, we assume that the minor splice isoform (ß) is responsible for the intrahepatic biliary network phenotypes observed in *nckap1l*^*lri35*^ mutant larvae. Consistently, injecting the RNA encoding the minor *nckap1l* isoform at least partially rescued the *nckap1l*^*lri35*^ mutant phenotype (Fig 4M–4O). Although specific binding domains of Nckap1l to other WRC proteins are not precisely determined yet, it will be important to identify binding partners that specifically bind to the Nckap1l ß specific C-terminus domain (Fig 2D), which is mutated by the *lri35* mutation. We detected strong Nckap1l expression in vascular endothelial cells in the liver (Figs 3A and S6), we did not observe overt differences in the vasculature of *nckap1l*^*lri35*^ mutant larvae (S8 Fig). However, in *nckap1l*^*lri90*^ mutant larvae at 6 dpf, although the penetrance is low, we observed disrupted intersegmental vessels (S8 Fig), suggesting that Nckap1 α might be predominantly required for vascular angiogenesis. It is not yet known whether a similar minor splice isoform of *NCKAP1L* exists in humans; however, the last exon of human *NCKAP1L* is large. It would be intriguing to clone the short splice form of *NCKAP1L* in rodents and humans to understand the clinical relevance of this finding. Indeed sequence data derived from patients with biliary atresia and other cholangiopathies should be reexamined focusing on mutations in the last exon of human *NCKAP1L* after the conventional stop codon, as our data potentially suggest that the genomic region currently recognized as the 3’ UTR could encode a previously unannotated minor protein isoform.

Nckap1l knockout mice exhibited a 25-fold increase in the number of circulating neutrophils [16]; however, in zebrafish larvae, the number of neutrophils remains constant in *nckap1l*^*lri90*^ mutant larvae at 5 dpf (S9 Fig), suggesting that *nckap1l* is not required for the initial differentiation of neutrophils in zebrafish. We did not observe neutrophil accumulation in the liver of *nckap1l*^*lri90*^ mutant larvae at 5 dpf, suggesting that liver inflammation might not happen at this stage. As we found that the *nckap1l*^*lri90*^ mutation genetically interacts with *Tg(tp1*:*cdkal1)*^*kl109*^ (Fig 5), in which the Cdk5 inhibitor is specifically expressed by biliary epithelial cells within the liver, we assume that *nckap1l* functions cell-autonomously in biliary epithelial cells to regulate actin remodeling for branching morphogenesis. This view is consistent with our observation that Nckap1l is expressed in biliary epithelial cells (Fig 3B). However, our data do not exclude the possibility that the biliary phenotype seen in *nckap1l* mutant larvae might be secondary to a phenotype in innate immune cells. We also observed that the *nckap1l*^*lri90*^ mutation genetically interacts with the Rac1 pathway (Fig 6). Since the WRC is known to act downstream of Rac1 [36], we assume that Rac1 regulates the intrahepatic biliary network branching morphogenesis at least in part through the WRC containing Nckap1l. These data suggest that Nckap1l might be working as a signaling hub integrating the Cdk5 and Rac1 pathways to regulate proper branching morphogenesis of the intrahepatic biliary network (Fig 6M).

In conclusion, we have identified a previously unannotated splice isoform of *nckap1l* that is necessary for the branching morphogenesis of the intrahepatic biliary network and actin dynamics in biliary epithelial cells. The fact that minor splice isoform of the gene, whose major isoform is known to regulate innate immune cell differentiation and migration, plays pivotal roles in biliary system formation further implies that the correlation between cholangiopathy and inflammation might be due to genes required for both processes.

## Materials and methods

### Zebrafish husbandry and transgenic lines

Zebrafish (*Danio rerio*) larvae were obtained from natural crosses of the wild-type AB/TL strain or heterozygous mutant fish. The following transgenic and mutant lines were used: WT(AB/TL), *nckap1l*^*lri35*^ (originally named *JW-1*.*10*), *nckap1l*^*lri90*^, *Tg(Tp1-MmHbb*:*EGFP)*^*um14*^, *Tg(tp1*:*cdkal1)*^*kl109*^, *Tg(lyz*:*EGFP)*^*nz117*^ [49], *Tg(kdrl*:*GFP)*^*s843*^ [50], *Tg(kdrl*:*RFP_CAAX)*^*y171*^ [51], and *Tg(fabp10*:RFP*-CAAX)*^*lri2*^ [8]. Animal husbandry methods for this specific study including the use of 12-month-old adult fish were approved by the Cleveland Clinic’s Institutional Animal Care and Use Committee.

### N-ethylnitrosourea (ENU) mutagenesis and screening procedure

The standard three-generation screen with analysis of mutant phenotypes in F3 larvae was conducted as previously described [52]. In brief, 20 adult male AB/TL zebrafish were exposed to 3 mM ENU at weekly intervals four times. The mutagenized males were crossed to *Tg(Tp1-MmHbb*:*EGFP)*^*um14*^ fish. Total 228 F2 families representing 332.32 genomes were screened for altered *Tg(Tp1-MmHbb*:*EGFP)*^*um14*^ expression in the liver at 5 dpf. The phenotype was confirmed and quantified by the custom algorithm [8] as described below. We only collected mutants that showed no overt physical appearance phenotype at 5 dpf. We have recovered 24 alleles, including *nckap1l*^*lri35*^, showing phenotypes in the intrahepatic biliary network from this screen. The recovered *nckap1l*^*lri35*^ allele was backcrossed to the original AB/TL strain for 9 generations before starting detailed phenotype analyses.

### Computational network structure analysis

Confocal z-stack data of *Tg(Tp1-MmHbb*:EGFP*)*^*um14*^ expression were obtained using a Leica SP5 confocal microscope. The z-step used on the images was 0.25 μm. We used Imaris 8.2 software (Bitplane) to digitally crop the image such that only EGFP expression from the intrahepatic biliary network remained for further analysis. The Liver Analysis Program 5.3 [8] was used for all computational network structure analyses. For all analyses, we confirmed the proper skeletal conversion by overlaying the original confocal image with the converted skeletal image as previously described [8].

### Sample preparation, RNA-seq and bioinformatics

Total RNA from 20 wild-type siblings and 20 *nckap1l*^*lri35*^ mutant larvae was collected at 5 dpf using the Qiagen RNeasy mini kit (Qiagen, Cat. 74104) according to the manufacturer’s instructions. cDNA for next-generation sequencing was synthesized by using the TruSeq standard total RNA kit (Illumina, 20020598) according to the manufacturer’s instructions. Using an Illumina HighSeq 2500, a total of 11.5 and 10.5 millions of paired-end 50-bp reads were obtained from wild-type and mutant cDNA, respectively. Raw reads were aligned to the zebrafish genome (GRCz10) using TopHat [27]. The *lri35* mutation was mapped to chromosome 11 by MMAPPR [24]. The NGS and analyzed data are available in the GEO repository at the NCBI (GSE153386).

### Nckap1l antibody production

A polyclonal antibody against zebrafish Nckap1l, ab805, was produced by Thermo Fisher Scientific according to their established production protocol. In brief, zebrafish Nckap1l peptides, “RINHIKKCFSDPKRRP”, were synthesized, and NZW SPF rabbits were immunized. Serum was collected at day 90 and used at a 1:1 dilution in blocking buffer for immunohistochemistry. This antibody is predicted to recognize both α and ß isoforms of Nckap1l.

### Immunohistochemistry and other staining

For immunohistochemistry, zebrafish larvae were fixed with 2% formaldehyde in PEM (0.1 M Pipes, 2 mM EGTA, and 1.0 mM MgSO_4_), and the skin and yolk were removed prior to staining. All antibody staining was performed on fixed larvae in 10% Triton X-100 (Thermo Fisher, 8511) and 20% fetal bovine serum (FBS) in phosphate-buffered saline (PBS). The following primary antibodies were used: anti-WAVE1 at a 1:200 dilution (Novusbio, NB100-92239), anti-Abi1 at a 1:200 dilution (Thermo Fisher, PA5 35337), anti-Spgp (Abcb11) at a 1:200 dilution (Kamiya Biomedical Company, PC-064), anti-HSPC300 at a 1:200 dilution (Santa Cruz, sc-390459), anti-beta-tubulin (Abcam, Cat. 6046) at a 1:5,000 dilution, and anti-Nckap1l (ab805) at a 1:1 dilution. The secondary antibody goat anti-rabbit IgG ALEXA FLUOR 568 (Invitrogen, A11036) was used at a 1:200 dilution. The following staining treatments were also used: ALEXA FLUOR 647 Phalloidin at a 1:10 dilution (Thermo Fisher, A22287; 300 units) and DAPI at a 1:2000 dilution (Life Technologies, D1306; 10 mg).

### Generation of the *nckap1l*^*lri90*^ allele

To create the *lri90* mutation, the following crRNA sequence was used: 5’-CUCCGCCAGUUUCAGCUGGUGUUUUAGAGCUAUGCU-3’, which was injected into wild-type fertilized eggs. The CRISPR/Cas9 complex was assembled according to the manufacturer’s instructions. In brief, 3 μL of 100 μM crRNA, 3 μL of 100 μM Alt-R CRISPR-Cas9 tracrRNA (Integrated DNA Technologies, 209702895), and 94 μL of nuclease free water were mixed. The mixture was then heated to 95°C. Two microliters of the mixture, 0.5 μL of Alt-R S.P. Cas9 Nuclease V3 (Integrated DNA Technologies, 209702894), 2 μL of phenol red, and 5 μL water were mixed and heated to 37°C before injection. We injected this solution into fertilized zebrafish eggs, and we subsequently established the *nckap1l*^*lri90*^ allele which disrupts both *nckap1l* α and ß isoforms.

### Liver size measurement

The liver size was measured based on the images of *Tg(fabp10*:RFP*-CAAX)*^*lri2*^ expression at 5 dpf utilizing the ImageJ software.

### *nckap1l* morpholino injection

*nckap1l* splice blocking morpholino (5’-AGCGGCTCCGCTCACCTTCTTGATG-3’) was designed and injected as previously described [38]. This morpholino is predicted to block proper splicing of both *nckap1l* α and ß isoforms.

### Genotyping of the *lri35* and *lri90* alleles

The following primers were used to genotype the *lri35* allele: forward primer that we named 17, 5’-GTCAGGATATGCTGGAGATGTG-3’; reverse primer that we named JW-genotyping_AR1, 5’-TGATCTGGATTCTGAAGAAGCCACTGA-3’. The *lri35* mutation induces an *MboII* site in the PCR product amplified by these primers. The following primers were used to genotype the *lri90* allele: forward primer that we named pam-F1, 5’-GATTAGAGAAAGCTGAGAGCGGAAGTG-3’; reverse primer that we named pam-R1, 5’-ACTGAGGACTTCAGAAGCGGCTCCGCT-3’. The *lri90* mutation eliminates the *PvuII* site in the PCR product amplified by these primers.

### Cloning of *nckap1l* isoforms and RNA synthesis for injection

*nckap1l* α was sub-cloned from a PCR product amplified from the cDNA of 5 dpf wild-type zebrafish larvae using the following primers: forward primer, 5’- CACACTCACCATGGCCTAC-3’; reverse primer, 5’-GACGTGTGATCTCCCTGATAAC-3’. *nckap1l* ß was PCR amplified using the following primers: forward primer, 5’-CACACTCACCATGGCCTAC-3’; reverse primer, 5’-CCTCACACACAGCGTAATGA-3’. The *nckap1l* isoforms were gel purified and sub-cloned using the TOPO TA Cloning Kit Dual Promoter (Life Technologies, 450640). *nckap1l* ß was sub-cloned into PCS2+. The *nckap1l* ß pCS2+ plasmids was digested with NotI-HF (New England BioLabs, R3189; 500 units), and the mRNA was synthesized using the mMessage SP6 kit (Invitrogen, AM1340; 25 reactions). *nckap1l* ß synthesized RNA was injected into wild-type eggs at 0–1 hpf at amounts of 160 pg per egg.

### PED6-based biliary system functional assay

PED6 treatment and measurement were performed as previously described [8]. In brief, zebrafish larvae obtained from heterozygous *nckap1l*^*lri90*^ mutant fish cross were soaked in PED6-containing media from 4 to 5 dpf, scored PED6 staining in the gallbladder as previously described [8], and genotyped.

### Real-time qPCR

Real-Time qPCR was performed as previously described [38]. We used following primers: *nckap1l α* (5’- GTCCTCTGTCCTACTCCAGCTC-3’ and 5’-TCTCTGTATGCGTTTCTGAGGA-3’); *nckap1l ß* (5’-TCCCGCTCGTCTGCTCACACTTCAGCC-3’ and 5’-GATTGATGTAAGCAGTGGTGTTG-3’); control *b2m* (5’-GCCTTCACCCCAGAGAAAGG-3’ and 5’-GCGGTTGGGATTTACATGTTG-3’). The ΔΔCt method using *b2m* as a reference was used for relative quantification.

### *In situ* hybridization

*In situ* hybridization was performed as previously described [38]. *nckap1l α* specific and *nckap1l α* and *ß* probes were synthesized using the PCR products amplified with the following primers as a template: *α* specific (5’-GGTGTTTGTGCAGATGGCGGGCTAC -3’ and 5’- CACCGATTTAGGTGACACTATAGgtagagtgtggtgatggtctgcgcgc-3’) and *α* and *ß* (5’-ATTGTGAATTTTGACCTAGACCAAGA-3’ and 5’-CACCGATTTAGGTGACACTATAGctgttcccgttggacagctcatacg-3’).

### Pharmacological treatments

Rac1 inhibitor (EMD Biosciences, Product #553502) was treated as previously described [37].

### Adult liver tissue processing and imaging

12-month-old adult fish were dissected to examine altered *Tg(Tp1-MmHbb*:EGFP*)*^*um14*^ expression in the liver. The liver lobe fixed in 2% FA in PEM was embedded in 2% GeneMate LowMelt Agarose (Cat. No. E-3126-25) in PBS. The agarose block was sliced at a thickness of 250 μm with Leica VT1000S vibratome. The slices were scanned on a Leica SP5 confocal microscope. *Tg(Tp1-MmHbb*:*EGFP)*^*um14*^ expression in the liver was scanned in a z-stack image, and the projected images were generated by Bitplane Imaris software.

### Statistics

For pairwise analysis, Student’s t-test was used to compare means assuming unequal variance. To compare three or more means, one-way ANOVA followed by Tukey’s HSD test was used.

## Supporting information

S1 Fig *Nckap1l* morpholino (MO) injection changes the branching pattern of the intrahepatic biliary network. (A and B) Representative physical appearance of control WT (A) and *nckap1l* MO-injected (B) larvae at 5 dpf. Lateral views. At 5 dpf, there is no significant difference in the physical appearance in *nckap1l* MO-injected larvae. (C and D) Projected confocal images of *Tg(Tp1-MmHbb*:EGFP*)*^*um14*^ expression in control WT (C) and *nckap1l* MO-injected (D) larvae at 5 dpf. GFP expression in the intrahepatic biliary network is shown in pseudocolored yellow. Ventral views, anterior to the top. (E and F) Skeletal representation of the intrahepatic biliary network in control WT (E) and *nckap1l* MO-injected (F) larvae computed based on *Tg(Tp1-MmHbb*:EGFP*)*^*um14*^ expression at 5 dpf. The end points (green), nodes (white), node-node connections (red), and node-end point connections (yellow) are colored separately. (G-L) Computational analysis-based measurements of the intrahepatic biliary network structures of control WT and *nckap1l* MO-injected larvae at 5 dpf. n = 6 for control WT and n = 5 for MO-injected larvae. (G) The total network volume of the intrahepatic biliary network marked by *Tg(Tp1-MmHbb*:EGFP*)*^*um14*^ expression in the liver. (H) The total network length of the intrahepatic biliary network. (I) The average thickness of the intrahepatic biliary network. (J) Total number of 3-way branching nodes existing in the intrahepatic biliary network. (K) Total number of 4-way branching nodes. (L) Total number of 5-or-more-way branching nodes. Error bars are standard deviation. *P<0.05, **P<0.01. n.s., not significant.
(TIF)

S2 Fig CRISPR/Cas9-mediated *nckap1l* knockout induces biliary phenotypes similar to those in *lri35* mutant larvae. (A) Cas9 protein guide RNA was designed against the first exon of the *nckap1l* gene. (B) The CRISPR/Cas9-derived *nckap1l*^*lri90*^ allele deletes 7 bp from the first exon of the *nckap1l* gene. Green box indicates the initial codon. (C) In *nckap1l*^*lri90*^ mutant larvae, the 7 bp deletion replaces the tyrosine (^3^Y) residue with a stop codon. (D and E) Representative physical appearance of WT (D) and *nckap1l*^*lri90*^ mutant (E) larvae at 5 dpf. Lateral views. At 5 dpf, there is no significant difference in physical appearance in *nckap1l*^*lri90*^ mutant larvae. (F and G) Projected confocal images of *Tg(Tp1-MmHbb*:EGFP*)*^*um14*^ expression in control WT (C) and *nckap1ll*^*ri90*^ mutant (D) larvae at 5 dpf. GFP expression in the intrahepatic biliary network is shown in pseudocolored yellow. Ventral views, anterior to the top. (H and I) Skeletal representation of the intrahepatic biliary network in WT (H) and *nckap1l*^*lri90*^ mutant (I) larvae computed based on *Tg(Tp1-MmHbb*:EGFP*)*^*um14*^ expression at 5 dpf. The end points (green), nodes (white), node-node connections (red), and node-end point connections (yellow) are colored separately. (J-R) Computational skeletal analysis-based measurements of the intrahepatic biliary network structures of control wild-type siblings (WT) and *nckap1l*^*lri90*^ mutant larvae at 5 dpf. n = 5 for WT and n = 7 for *nckap1l*^*lri90*^ mutant larvae. (J) The total network volume of the intrahepatic biliary network marked by *Tg(Tp1-MmHbb*:EGFP*)*^*um14*^ expression in the liver. (K) The total network length of the intrahepatic biliary network. (L) The average thickness of the intrahepatic biliary network. (M) The ratio of 3-way branching nodes per all nodes shown as a percentage. (N) The ratio of 4-way branching nodes per all nodes shown as a percentage. (O) The ratio of 5-or-more-way branching nodes per all nodes shown as a percentage. (P) Total number of nodes. (Q) Total number of connections. (R) Total number of unconnected branches. (S) Percentage of larvae showing high, low and no PED6 fluorescence in the gallbladder of WT or *nckap1l*^*lri90*^ mutant (MUT) larvae at 5 dpf (Materials and Methods). In *nckap1l*^*lri90*^ mutant larvae, PED6 transport to the gallbladder is reduced, suggesting that the functionality of the biliary network is impaired. (T and U) Projected confocal images of the adult liver visualized for *Tg(Tp1-MmHbb*:*EGFP)*^*um14*^ expression in wild-type (T) and *nckap1l*^*lri90*^ mutant (U) 12-month-old fish. The 12.6 um thickness cross sections of the liver were projected and shown. The *Tg(Tp1-MmHbb*:*EGFP)*^*um14*^ expressing intrahepatic biliary network appears to be thinner and less dense in *nckap1l*^*lri90*^ mutant fish than in wild-type fish. However, as previously reported [46], the variability in adult biliary network phenotype is high. n>10 for WT, and n = 3 for *nckap1l*^*lri90*^ fish. *P<0.05, **P<0.01, and ***P<0.001. n.s., not significant.
(TIF)

S3 Fig The *nckap1l*^*lri90*^ mutant failed to complement the *lri35* mutant, indicating that these two mutations are affecting the same gene. Homozygous *nckap1l*^*lri90*^ mutant fish were crossed to heterozygous *lri35* mutant fish, and approximately 50% (20/43) of larvae showed a phenotype indistinguishable from *nckap1l*^*lri90*^ mutant larvae. (A and B) Representative physical appearance of heterozygous *nckap1l*^*lri90*^ (A) and *nckap1l*^*lri90/lri35*^ compound heterozygous (B) larvae at 5 dpf. (C and D) Lateral views of *Tg(Tp1-MmHbb*:EGFP*)*^*um14*^ expression in heterozygous *nckap1l*^*lri90*^ (C) and *nckap1l*^*lri90/lri35*^ compound heterozygous (D) larvae. In *nckap1l*^*lri90/lri35*^ compound heterozygous larvae, the intrahepatic biliary network is reduced as seen in *nckap1l*^*lri90*^ and *lri35* mutant larvae, indicating that these two alleles fail to complement.
(TIF)

S4 Fig Sanger sequencing confirms a one-nucleotide insertion in the *nckap1l* gene. *nckap1l* cDNA isolated from wild-type (WT) and *nckap1l*^*lri35*^ mutant larvae was sequenced in the 3’ to 5’ direction. The green box indicates the inserted thymine nucleotide, which induces a frameshift specifically in the minor ß splice isoform of *nckap1l*.
(TIF)

S5 Fig *nckap1l* expression in wild-type larvae at 5 dpf. (A) qPCR analysis of *nckap1l* α mRNA expression levels in wild-type control and *nckap1l*^*lri35*^ mutant larvae at 5 dpf. (B) qPCR analysis of *nckap1l* ß mRNA expression levels in wild-type control and *nckap1l*^*lri35*^ mutant larvae at 5 dpf. The averages of at least three independent experiments are shown. *nckap1l* ß mRNA expression level is slightly down-regulated while *nckap1l* α mRNA expression level remains constant in *nckap1l*^*lri35*^ mutant larvae. *P<0.05, n.s., not significant; error bars indicate standard deviation. (C and D) *nckap1l* mRNA expression in wild-type larvae was examined by *in situ* hybridization at 5 dpf. Since the entire *nckap1l ß* gene sequence is part of the ORF and UTR of the *nckap1l* α gene, we were not able to design the *nckap1l ß*-specific RNA probe. Instead, we used two different RNA probes for *in situ* hybridization; one specifically recognizes *nckap1l* α and the other recognizes both *nckap1l* α and *ß* isoforms. (C) *nckap1l* expression in wild-type larvae at 5 dpf. The RNA probe that recognizes both α and *ß* isoforms was used. (D) *nckap1l* expression in wild-type larvae at 5 dpf. The RNA probe that recognizes only the α isoform was used. Black broken lines outline the liver position in C’ and D’. *nckap1l* is expressed widely including in the vertebrate, intestine and swim bladder. In the liver, *nckap1l ß* appears to be expressed more, as the α and *ß* isoform probe (C’) shows a stronger signal than that of the α isoform specific probe (D’).
(TIF)

S6 Fig Nckap1l is expressed in endothelial cells in the liver. Z-plane confocal section of the liver showing anti-Nckap1l staining (Red) at 5 dpf (A). Overlay with vascular endothelial cell marker *Tg(kdrl*:*GFP)*^*s843*^ is shown separately in (B). Nckap1l is predominantly expressed in endothelial cells (blue arrowheads) in the liver. Punctate Nckap1l is also observed in biliary epithelial cells (white arrows). Ventral views, anterior to the top. EC, endothelial cells.
(TIF)

S7 Fig Western blotting of WAVE regulatory complex (WRC) proteins. Nckap1l, Abi1, and HSPC300 expression levels in wild-type, *nckap1l*^*lri90*^ mutant, and *nckap1l*^*lri35*^ mutant larvae were analyzed by western blotting at 5 dpf. Whole-body homogenates of 5 dpf larvae were used. All WRC protein levels were reduced in *nckap1l*^*lri35*^ mutant larvae, suggesting that WRC proteins were degraded. Tubulin blotting was for loading control. These experiments were repeated three times with similar results.
(TIF)

S8 Fig Formation of intersegmental vessels in *nckap1l* mutant larvae. (A-D) Lateral views of the trunk in wild-type (A), *nckap1l*^*lri35*^ (B), and *nckap1l*^*lri90*^ mutant (C and D) *Tg(kdrl*:*RFP_CAAX)*^*y171*^ larvae at 6 dpf. In all mutant larvae examined (n = 24 from three independent crosses), we did not observe any overt blood vessel phenotype in *nckap1l*^*lri35*^ mutant larvae at 6 dpf (B). In *nckap1l*^*lri90*^ mutant larvae at 6 dpf, the majority of mutant larvae (n = 27/45 from three independent crosses) show no overt blood vessel phenotype (C). However, we consistently observed disrupted intersegmental vessels (D) in some *nckap1l*^*lri90*^ mutant larvae (n = 18/45). These data suggest that although the phenotype penetrance is low, the *nckap1l*^*lri90*^ mutation can induce a specific morphological phenotype in the blood vessels.
(TIF)

S9 Fig The number of neutrophils is unchanged in *nckap1l*^*lri90*^ mutant larvae at 5 dpf. (A and B) Lateral views of the trunk in wild-type (A) and *nckap1l*^*lri90*^ mutant (B) *Tg(lyz*:*EGFP)*^*nz117*^ larvae at 5 dpf. The number of *Tg(lyz*:*EGFP)*^*nz117*^-expressing neutrophils is not changed in *nckap1l*^*lri90*^ mutant larvae. (C and D) Lateral views of the tail in wild-type (C) and *nckap1l*^*lri90*^ mutant (D) *Tg(lyz*:*EGFP)*^*nz117*^ larvae at 5 dpf. Lateral views, anterior to the left. (E) The number of *Tg(lyz*:*EGFP)*^*nz117*^*-*expressing neutrophils in the tail of wild-type and *nckap1l*^*lri90*^ mutant larvae.
(TIF)
