# Supplementary figures and images for: NCK-associated protein 1 like (nckap1l) minor splice variant regulates intrahepatic biliary network morphogenesis
Source: PLoS Genet. 2021 Mar 19;17(3):e1009402. doi: 10.1371/journal.pgen.1009402 (PMC8032155; doi:10.1371/journal.pgen.1009402)

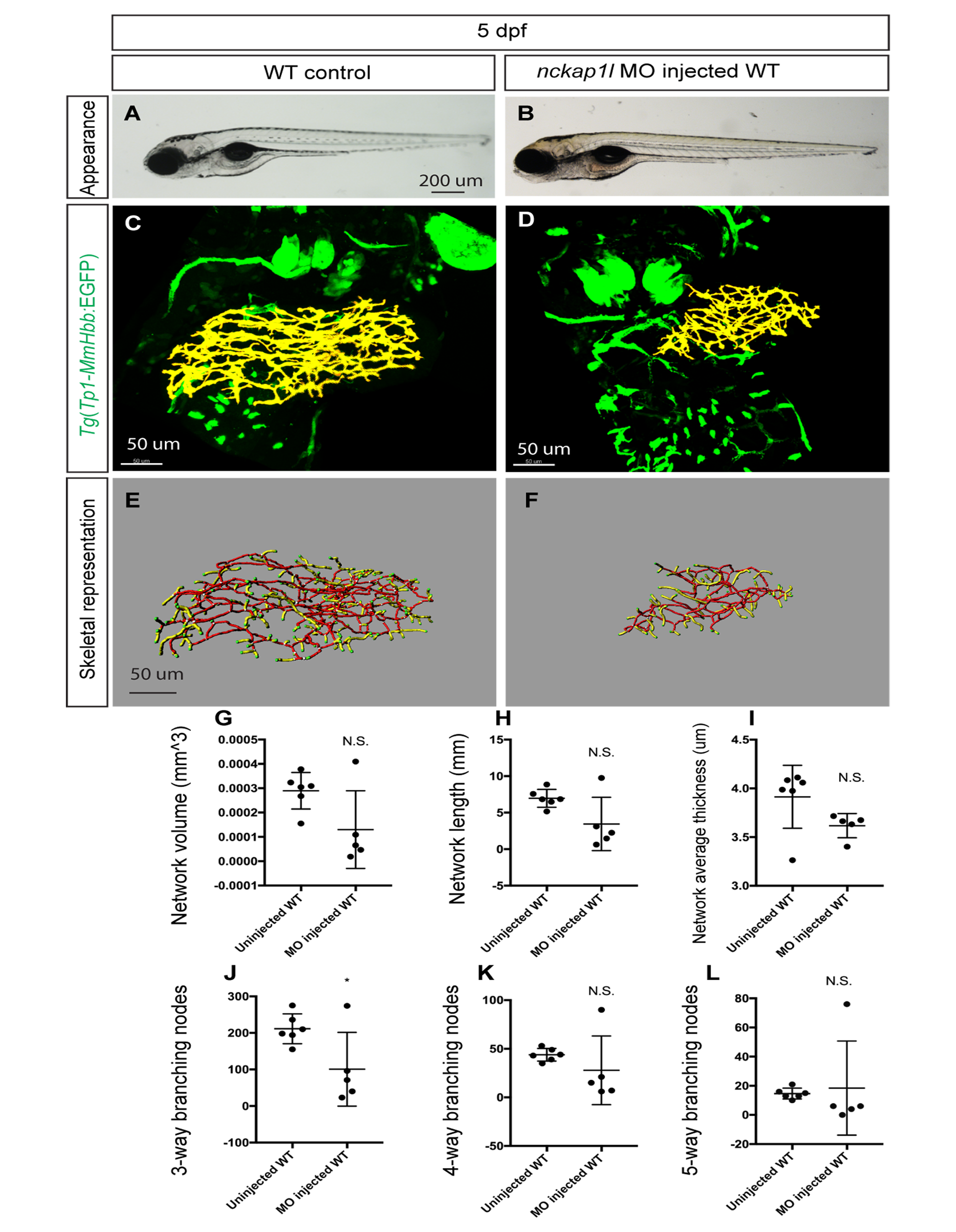

Supplement: S1 Fig — (A and B) Representative physical appearance of control WT (A) and nckap1l MO-injected (B) larvae at 5 dpf. Lateral views. At 5 dpf, there is no significant difference in the physical appearance in nckap1l MO-injected larvae. (C and D) Projected confocal images of Tg(Tp1-MmHbb:EGFP)um14 expression in control WT (C) and nckap1l MO-injected (D) larvae at 5 dpf. GFP expression in the intrahepatic biliary network is shown in pseudocolored yellow. Ventral views, anterior to the top. (E and F) Skeletal representation of the intrahepatic biliary network in control WT (E) and nckap1l MO-injected (F) larvae computed based on Tg(Tp1-MmHbb:EGFP)um14 expression at 5 dpf. The end points (green), nodes (white), node-node connections (red), and node-end point connections (yellow) are colored separately. (G-L) Computational analysis-based measurements of the intrahepatic biliary network structures of control WT and nckap1l MO-injected larvae at 5 dpf. n = 6 for control WT and n = 5 for MO-injected larvae. (G) The total network volume of the intrahepatic biliary network marked by Tg(Tp1-MmHbb:EGFP)um14 expression in the liver. (H) The total network length of the intrahepatic biliary network. (I) The average thickness of the intrahepatic biliary network. (J) Total number of 3-way branching nodes existing in the intrahepatic biliary network. (K) Total number of 4-way branching nodes. (L) Total number of 5-or-more-way branching nodes. Error bars are standard deviation. *P<0.05, **P<0.01. n.s., not significant. (TIF) [file pgen.1009402.s001.tif]

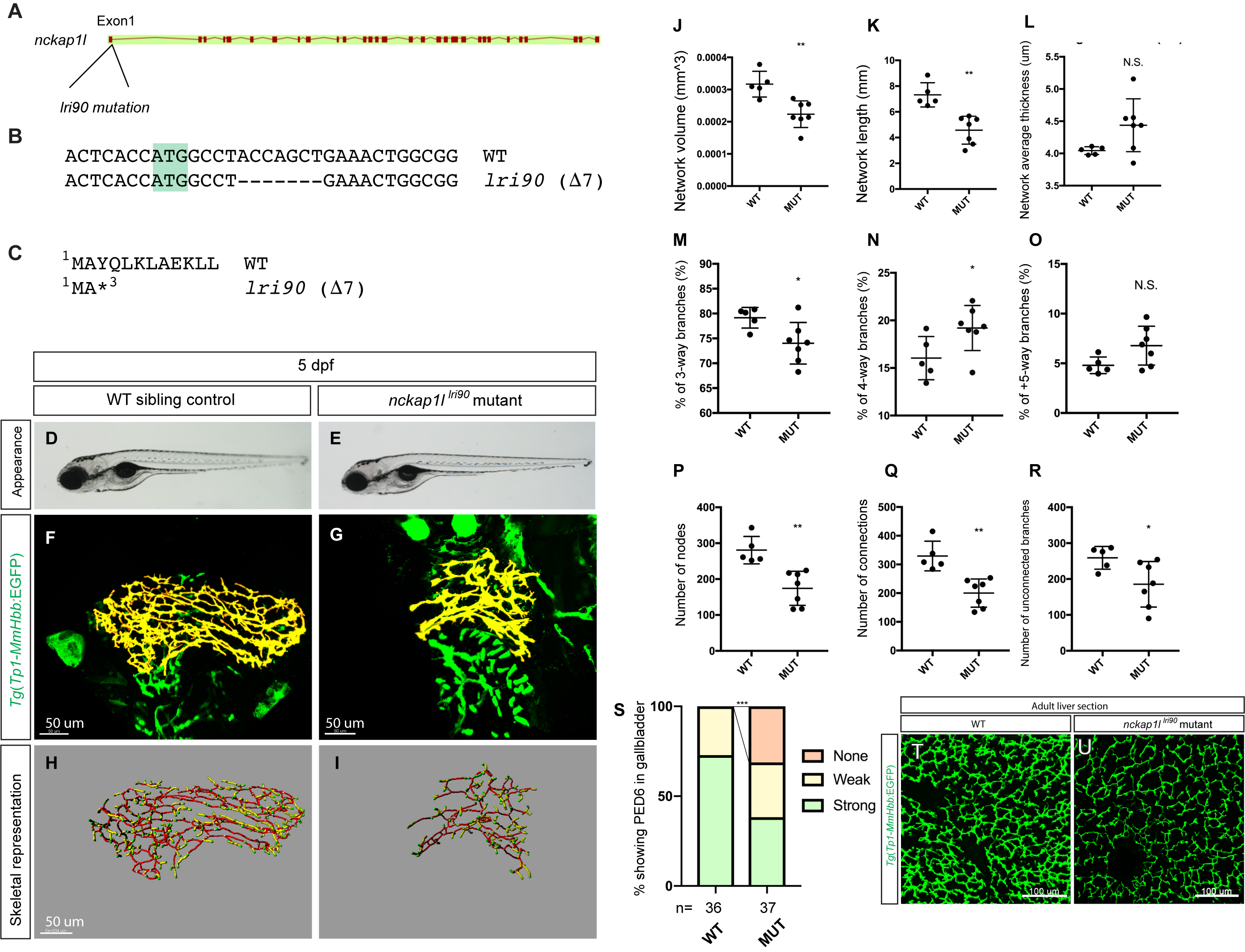

Supplement: S2 Fig — (A) Cas9 protein guide RNA was designed against the first exon of the nckap1l gene. (B) The CRISPR/Cas9-derived nckap1llri90 allele deletes 7 bp from the first exon of the nckap1l gene. Green box indicates the initial codon. (C) In nckap1llri90 mutant larvae, the 7 bp deletion replaces the tyrosine (3Y) residue with a stop codon. (D and E) Representative physical appearance of WT (D) and nckap1llri90 mutant (E) larvae at 5 dpf. Lateral views. At 5 dpf, there is no significant difference in physical appearance in nckap1llri90 mutant larvae. (F and G) Projected confocal images of Tg(Tp1-MmHbb:EGFP)um14 expression in control WT (C) and nckap1llri90 mutant (D) larvae at 5 dpf. GFP expression in the intrahepatic biliary network is shown in pseudocolored yellow. Ventral views, anterior to the top. (H and I) Skeletal representation of the intrahepatic biliary network in WT (H) and nckap1llri90 mutant (I) larvae computed based on Tg(Tp1-MmHbb:EGFP)um14 expression at 5 dpf. The end points (green), nodes (white), node-node connections (red), and node-end point connections (yellow) are colored separately. (J-R) Computational skeletal analysis-based measurements of the intrahepatic biliary network structures of control wild-type siblings (WT) and nckap1llri90 mutant larvae at 5 dpf. n = 5 for WT and n = 7 for nckap1llri90 mutant larvae. (J) The total network volume of the intrahepatic biliary network marked by Tg(Tp1-MmHbb:EGFP)um14 expression in the liver. (K) The total network length of the intrahepatic biliary network. (L) The average thickness of the intrahepatic biliary network. (M) The ratio of 3-way branching nodes per all nodes shown as a percentage. (N) The ratio of 4-way branching nodes per all nodes shown as a percentage. (O) The ratio of 5-or-more-way branching nodes per all nodes shown as a percentage. (P) Total number of nodes. (Q) Total number of connections. (R) Total number of unconnected branches. (S) Percentage of larvae showing high, low and no PED6 fluoresc [file pgen.1009402.s002.tif]

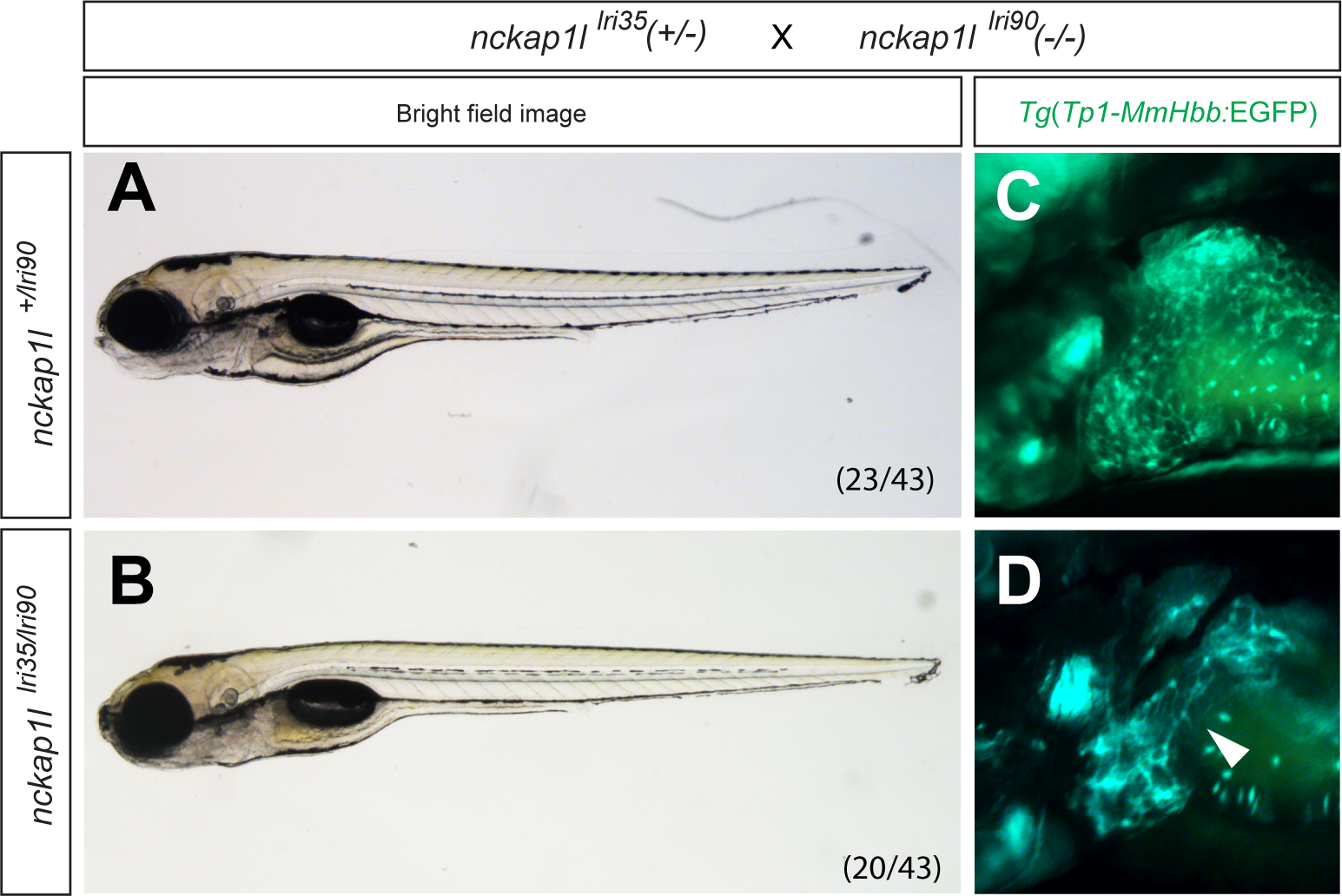

Supplement: S3 Fig — Homozygous nckap1llri90 mutant fish were crossed to heterozygous lri35 mutant fish, and approximately 50% (20/43) of larvae showed a phenotype indistinguishable from nckap1llri90 mutant larvae. (A and B) Representative physical appearance of heterozygous nckap1llri90 (A) and nckap1llri90/lri35 compound heterozygous (B) larvae at 5 dpf. (C and D) Lateral views of Tg(Tp1-MmHbb:EGFP)um14 expression in heterozygous nckap1llri90 (C) and nckap1llri90/lri35 compound heterozygous (D) larvae. In nckap1llri90/lri35 compound heterozygous larvae, the intrahepatic biliary network is reduced as seen in nckap1llri90 and lri35 mutant larvae, indicating that these two alleles fail to complement. (TIF) [file pgen.1009402.s003.tif]

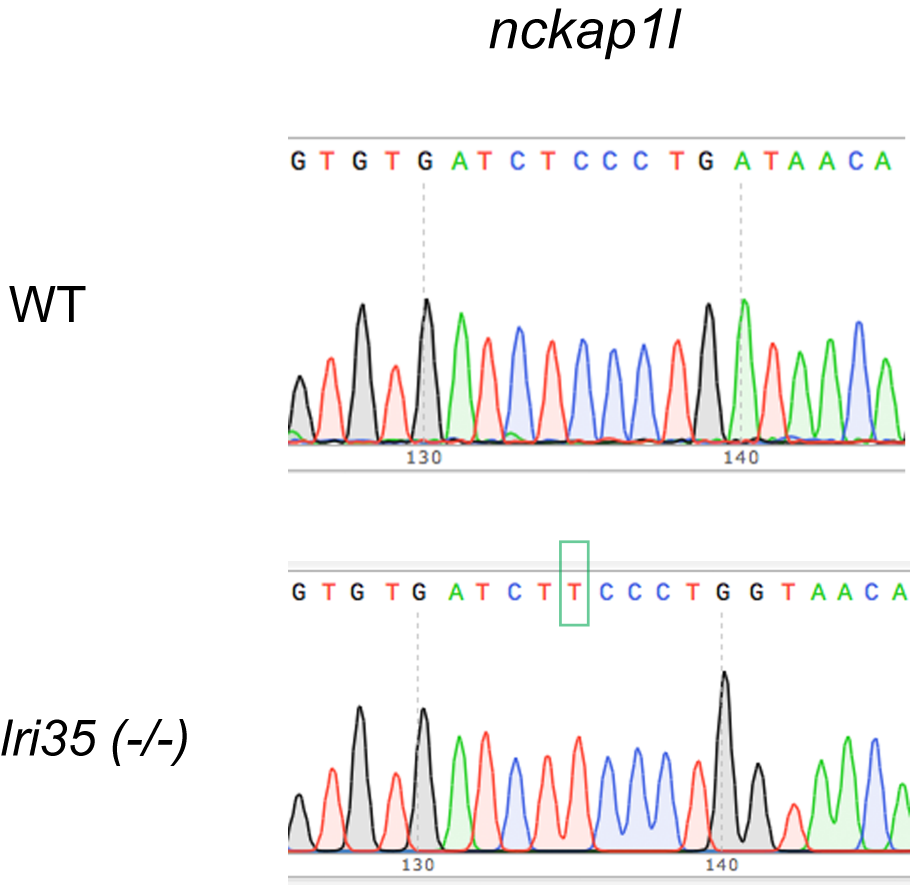

Supplement: S4 Fig — nckap1l cDNA isolated from wild-type (WT) and nckap1llri35 mutant larvae was sequenced in the 3’ to 5’ direction. The green box indicates the inserted thymine nucleotide, which induces a frameshift specifically in the minor ß splice isoform of nckap1l. (TIF) [file pgen.1009402.s004.tif]

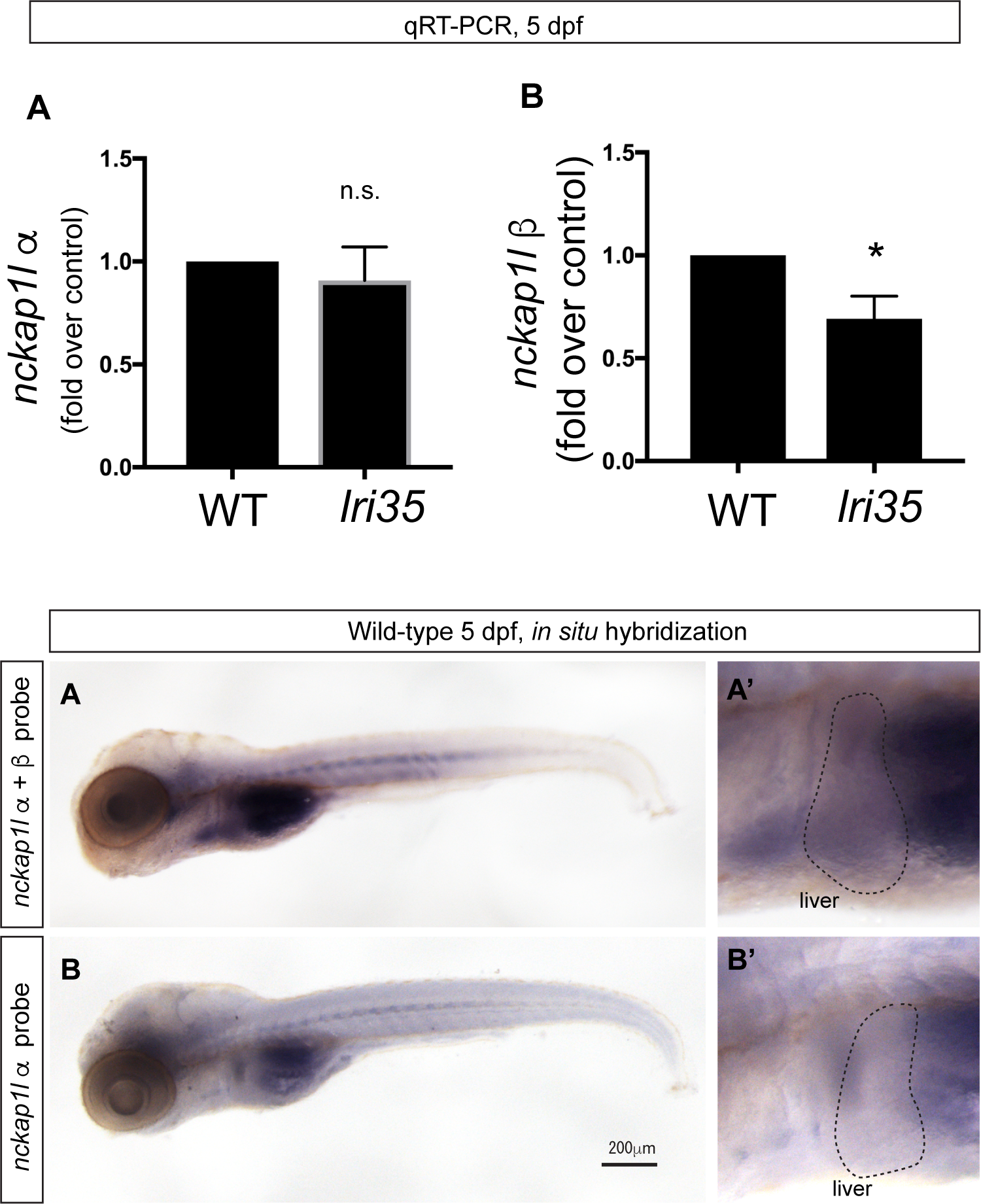

Supplement: S5 Fig — (A) qPCR analysis of nckap1l α mRNA expression levels in wild-type control and nckap1llri35 mutant larvae at 5 dpf. (B) qPCR analysis of nckap1l ß mRNA expression levels in wild-type control and nckap1llri35 mutant larvae at 5 dpf. The averages of at least three independent experiments are shown. nckap1l ß mRNA expression level is slightly down-regulated while nckap1l α mRNA expression level remains constant in nckap1llri35 mutant larvae. *P<0.05, n.s., not significant; error bars indicate standard deviation. (C and D) nckap1l mRNA expression in wild-type larvae was examined by in situ hybridization at 5 dpf. Since the entire nckap1l ß gene sequence is part of the ORF and UTR of the nckap1l α gene, we were not able to design the nckap1l ß-specific RNA probe. Instead, we used two different RNA probes for in situ hybridization; one specifically recognizes nckap1l α and the other recognizes both nckap1l α and ß isoforms. (C) nckap1l expression in wild-type larvae at 5 dpf. The RNA probe that recognizes both α and ß isoforms was used. (D) nckap1l expression in wild-type larvae at 5 dpf. The RNA probe that recognizes only the α isoform was used. Black broken lines outline the liver position in C’ and D’. nckap1l is expressed widely including in the vertebrate, intestine and swim bladder. In the liver, nckap1l ß appears to be expressed more, as the α and ß isoform probe (C’) shows a stronger signal than that of the α isoform specific probe (D’). (TIF) [file pgen.1009402.s005.tif]

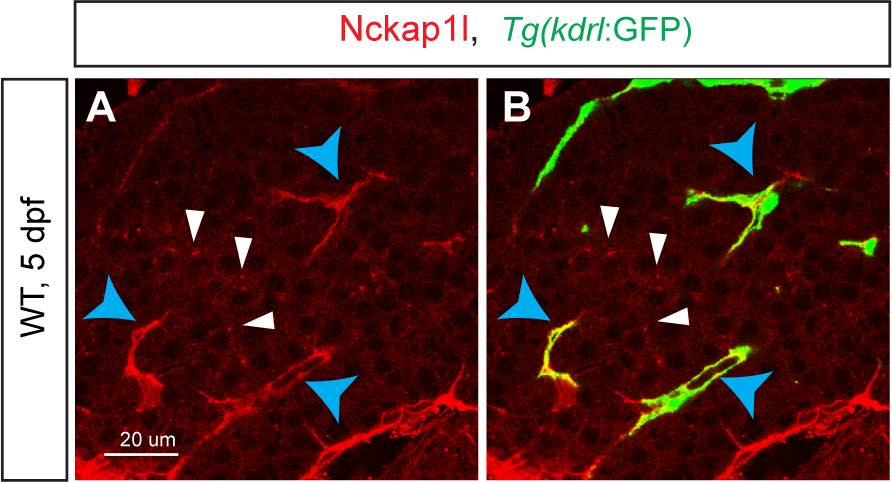

Supplement: S6 Fig — Z-plane confocal section of the liver showing anti-Nckap1l staining (Red) at 5 dpf (A). Overlay with vascular endothelial cell marker Tg(kdrl:GFP)s843 is shown separately in (B). Nckap1l is predominantly expressed in endothelial cells (blue arrowheads) in the liver. Punctate Nckap1l is also observed in biliary epithelial cells (white arrows). Ventral views, anterior to the top. EC, endothelial cells. (TIF) [file pgen.1009402.s006.tif]

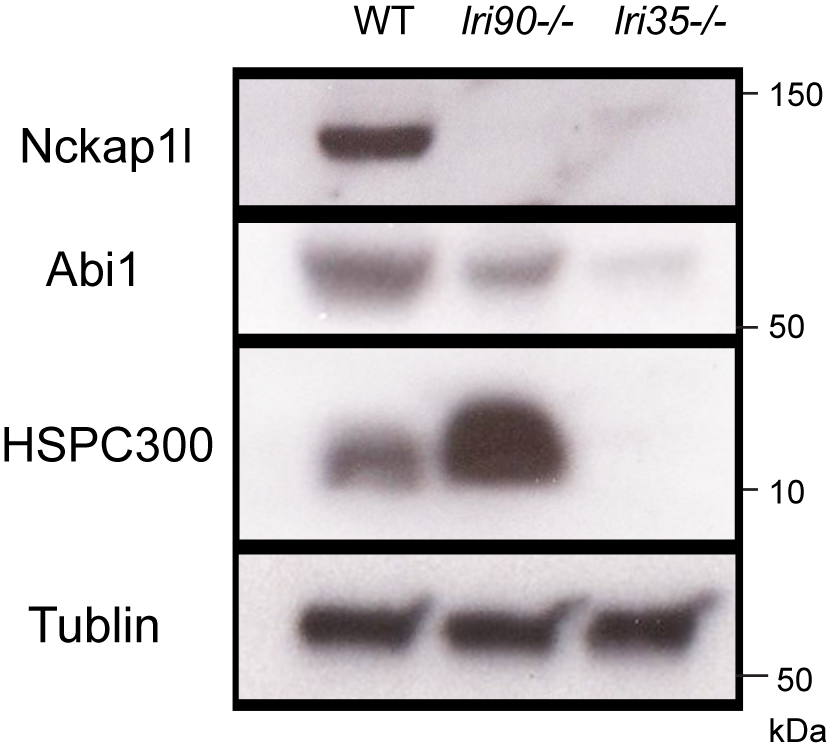

Supplement: S7 Fig — Nckap1l, Abi1, and HSPC300 expression levels in wild-type, nckap1llri90 mutant, and nckap1llri35 mutant larvae were analyzed by western blotting at 5 dpf. Whole-body homogenates of 5 dpf larvae were used. All WRC protein levels were reduced in nckap1llri35 mutant larvae, suggesting that WRC proteins were degraded. Tubulin blotting was for loading control. These experiments were repeated three times with similar results. (TIF) [file pgen.1009402.s007.tif]

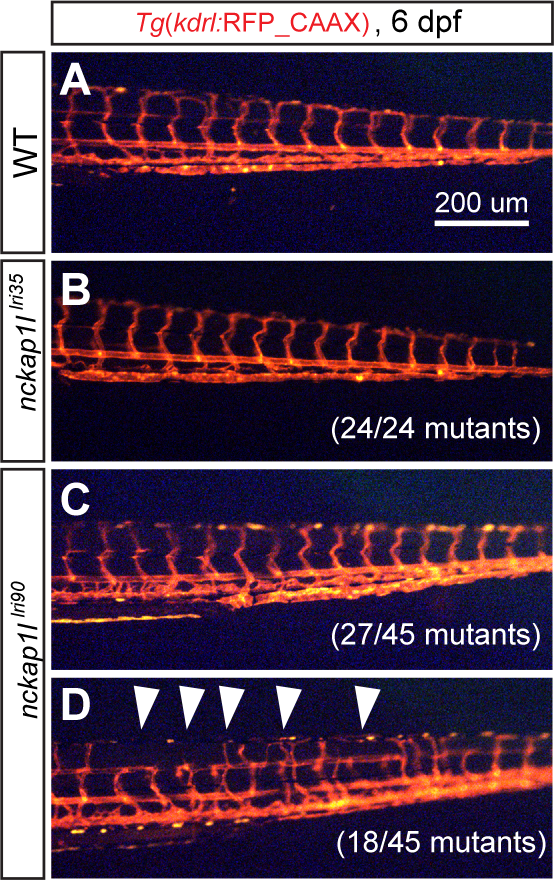

Supplement: S8 Fig — (A-D) Lateral views of the trunk in wild-type (A), nckap1llri35 (B), and nckap1llri90 mutant (C and D) Tg(kdrl:RFP_CAAX)y171 larvae at 6 dpf. In all mutant larvae examined (n = 24 from three independent crosses), we did not observe any overt blood vessel phenotype in nckap1llri35 mutant larvae at 6 dpf (B). In nckap1llri90 mutant larvae at 6 dpf, the majority of mutant larvae (n = 27/45 from three independent crosses) show no overt blood vessel phenotype (C). However, we consistently observed disrupted intersegmental vessels (D) in some nckap1llri90 mutant larvae (n = 18/45). These data suggest that although the phenotype penetrance is low, the nckap1llri90 mutation can induce a specific morphological phenotype in the blood vessels. (TIF) [file pgen.1009402.s008.tif]

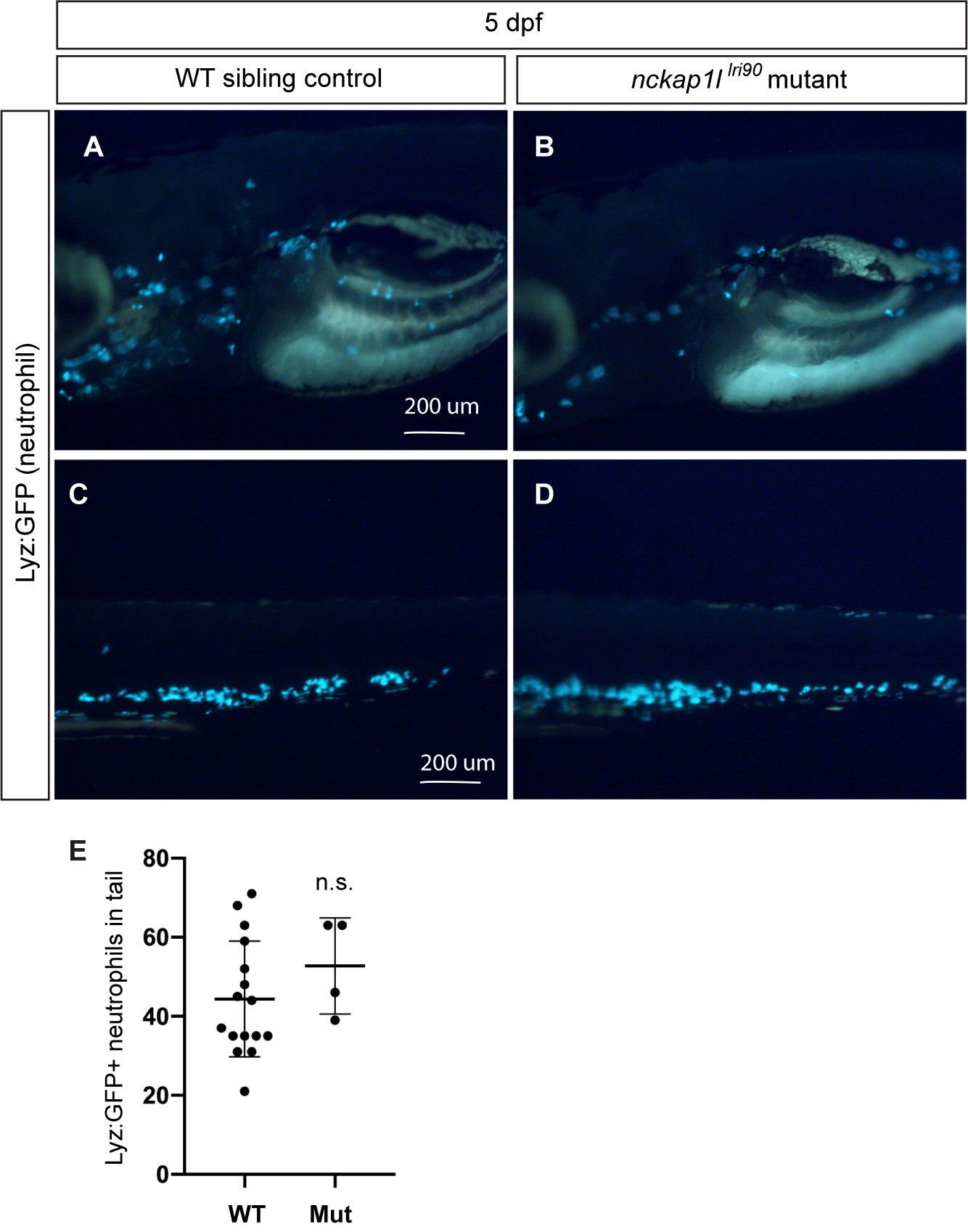

Supplement: S9 Fig — (A and B) Lateral views of the trunk in wild-type (A) and nckap1llri90 mutant (B) Tg(lyz:EGFP)nz117 larvae at 5 dpf. The number of Tg(lyz:EGFP)nz117-expressing neutrophils is not changed in nckap1llri90 mutant larvae. (C and D) Lateral views of the tail in wild-type (C) and nckap1llri90 mutant (D) Tg(lyz:EGFP)nz117 larvae at 5 dpf. Lateral views, anterior to the left. (E) The number of Tg(lyz:EGFP)nz117-expressing neutrophils in the tail of wild-type and nckap1llri90 mutant larvae. (TIF) [file pgen.1009402.s009.tif]
